# Author Correction: Magnetic field screening in hydrogen-rich high-temperature superconductors

**DOI:** 10.1038/s41467-023-40837-2

**Published:** 2023-09-01

**Authors:** V. S. Minkov, S. L. Bud’ko, F. F. Balakirev, V. B. Prakapenka, S. Chariton, R. J. Husband, H. P. Liermann, M. I. Eremets

**Affiliations:** 1 Max Planck Institute for Chemistry, Hahn Meitner Weg 1, 55128 Mainz, Germany; 2 Ames Laboratory, U.S. Department of Energy, Iowa State University, Ames, IA 50011 USA; 3 Department of Physics and Astronomy, Iowa State University, Ames, IA 50011 USA; 4 Los Alamos National Laboratory, Los Alamos, NM 87545 USA; 5 Center for Advanced Radiation Sources, University of Chicago, 5640 South Ellis Avenue, Chicago, IL 60637 USA; 6 Photon Science, DESY, Notkestrasse 85, 22607 Hamburg, Germany

**Keywords:** Superconducting properties and materials, Condensed-matter physics

Correction to: *Nature Communications* 10.1038/s41467-022-30782-x, published online 09 June 2022

The original version of this Article contained an error in the caption of Figure 3, which omitted relevant information and incorrectly read:

‘**a**, **b** Virgin curves of the *M*(*H*) magnetization data for the *Im-3m*-H_3_S phase at *P*_*S*_ = 155 ± 5 GPa and the *Fm-3m*-LaH_10_ phase at *P*_*S*_ = 130 ± 8 GPa at selected temperatures. The curves were superimposed for a better representation; so the linear trend of *M*(*H*) dependences coincides for measurements at different temperatures.’

The corrected version is:

‘**a**, **b** Magnetic moment associated with the penetration of the applied magnetic field into the *Im-3m*-H_3_S phase at *P*_*S*_ = 155 ± 5 GPa and the *Fm-3m*-LaH_10_ phase at *P*_*S*_ = 130 ± 8 GPa based on virgin curves of the *M(H)* magnetization data at selected temperatures. The curves were superimposed by performing linear transformations for a better representation. A linear background, defined as a straight line connecting *M*(*H* = 0 T) and *M*(*H* = 1 T) at corresponding temperature, was subtracted. After that the data were normalized to *H* = 15 mT data so that to have the same initial linear *M(H)* slope.’

This has been corrected in both the PDF and HTML versions of the Article.

Secondly, the original version of this Article contained an error in the third paragraph of the section ‘Magnetization measurements’ in the Methods, which incorrectly read:

‘The value of *H*_*p*_, at which an applied magnetic field starts to penetrate into the sample, was determined from the onset of the evident deviation of the *M*(*H*) from the linear dependence. We extrapolated *H*_*p*_(*T*) to lower temperatures using the equation…’

The corrected version is:

‘The value of *H*_*p*_, at which an applied magnetic field starts to penetrate into the sample, was determined from the onset of the evident deviation of the *M*(*H*) from the linear dependence. We note that the raw magnetization curves include the significant diamagnetic response of the miniature high-pressure cell, which increases with the applied magnetic field (see Fig. 3e, f and Supplementary Figs. S10 and S11). To better illustrate the determination of *H*_*p*_ in Figs. 3a and 3b, we have subtracted a linear background from the measured *M(H)* magnetization data. This linear background was determined as the straight line connecting two endpoints: the magnetic moment value at *H* = 0 T (the starting point of measurements) and the magnetic moment value at *H* = 1 T (the highest value of the applied magnetic field) (see Supplementary Fig. S12). Subsequently, we performed additional linear transformations so that the curves have the same initial linear *M(H)* slope. Importantly, these linear manipulations do not affect the onset of the deviation of the *M(H)* virgin curve from the linear dependence. We extrapolated *H*_*p*_(*T*) to lower temperatures using the equation…’

This has been corrected in both the PDF and HTML versions of the Article.

Thirdly, the original version of the Supplementary Information associated with this Article omitted Supplementary Fig. S12. The HTML has been updated to include a corrected version of the Supplementary Information.

### Supplementary information

Updated Supplementary Information

