# Addendum: Magnetic field screening in hydrogen-rich high-temperature superconductors

**DOI:** 10.1038/s41467-026-73705-w

**Published:** 2026-10-06

**Authors:** V. S. Minkov, E. F. Talantsev, S. L. Bud’ko, F. F. Balakirev, M. I. Eremets

**Affiliations:** 1 Max Planck Institute for Chemistry; Hahn Meitner Weg 1, Mainz, 55128 Germany; 2 M.N. Mikheev Institute of Metal Physics, Ural Branch of the Russian Academy of Sciences, S. Kovalevskoy St 18, Ekaterinburg, 620108 Russian Federation; 3 NANOTECH Centre, Ural Federal University, Mira St 19, Ekaterinburg, 620002 Russian Federation; 4 Ames National Laboratory, U.S. Department of Energy, Iowa State University, Ames, IA 50011 USA; 5 Department of Physics and Astronomy, Iowa State University, Ames, IA 50011 USA; 6 Los Alamos National Laboratory, Los Alamos, NM 87545 USA

Addendum to: *Nature Communications* https://doi.org/10.1038/s41467-022-30782-x, published online 09 June 2022

In our paper^1^, we studied the magnetic response of H_3_S and LaH_10_ superconductors to an applied magnetic field using Superconducting Quantum Interference Device (SQUID) magnetometry. Prof. Hirsch, in his Matters Arising entitled “On the Author Correction to “Magnetic field screening in hydride superconductors” Nat Commun 15, 8144 (2024)” and in the *Comment*^2^, questioned the averaging procedure applied to the magnetization data and raised broader doubts about the superconducting nature of high-*T*_*c*_ hydrides. We accept that the original publication did not sufficiently describe the data processing steps, specifically the smoothing, averaging, and filtering procedures applied to the measured magnetic moment *M(H*_*appl*_*,T)* datasets used to determine the penetration field *H*_*p*_. Due to the SQUID magnetometer’s dual current source configuration, magnetization measurements on our micrometer-scale samples exhibit an increased noise level above approximately 30 mT. To extract values of *H*_*p*_ from these high-noise datasets, we applied standard mathematical smoothing/filtering techniques. In addition, here we describe an alternative method to determine *H*_*p*_ without any data processing, based on a methodology commonly used for extracting critical currents in superconductors^3^ from *V(I)* datasets. This approach was recently shown to be effective for *DC* magnetization data^4^. The *H*_*p*_ values of the present analysis are consistent with those published in our original work^1^. We emphasize that the *Comment*^2^ concerns the secondary matter of estimating Ginzburg-Landau parameter, not the fundamental evidence for superconductivity in hydride superconductors. Our full raw magnetization datasets – featuring both virgin and hysteretic *M(H*_*appl*_*,T)* curves – clearly show characteristic superconducting behavior below *T*_*c*_, and the absence of hysteresis above it. These datasets are openly accessible via the Open Science Framework (https://osf.io/7wqxb/), allowing independent evaluation of our analysis.

Despite the challenges of probing and studying superconductivity in samples only tens of micrometers in size, confined within diamond anvil cells under extreme pressures, the existence of superconductivity in hydrogen-rich compounds has been firmly established by various independent experimental techniques and independent research teams^5^. The most prominent of these compounds have demonstrated *T*_*c*_ values reaching approximately 203 K in H_3_S^6–9^, 245 K in YH_9_^10,11^ and 250 K in LaH_10_^12–17^.

Hydrogen-rich superconductors exhibit all necessary hallmarks of superconductivity, which have been probed under megabar pressures, and different research groups have well reproduced their properties^5^. High-temperature superconductivity at high pressures is evidenced by:

1. Zero electrical resistivity below *T*_*c*_, or at least orders of magnitude lower than that of any known metal;
2. The Meissner state, involving the expulsion of an applied magnetic field from the sample upon cooling below *T*_*c*_;
3. The characteristic complex response of a superconductor to applied magnetic fields, which differs between type-I and type-II superconductors. For type-II superconductors, several phenomena are associated with the penetration of magnetic fields into the sample and include trapped magnetic flux and its creep, vortex lattice melting, a decrease in *T*_*c*_ with an increase in an applied magnetic field, and the suppression of superconductivity at magnetic fields above the upper critical field *H*_*c2*_;
4. Superconducting energy gap indicating the collective nature of the charge carriers^18^;
5. Pronounced isotope effect (hydrides vs deuterides) demonstrating the involvement of the crystal lattice in phonon-mediated superconductivity.

Magnetic susceptibility measurements^6^ have recently been significantly improved for megabar high-pressure conditions^5^. Currently, three techniques are used for magnetic measurements at high pressures: a double modulation *AC* technique using a system of coils^19,20^, SQUID magnetometry^6,21^, and nitrogen-vacancy (NV) color centers implanted in diamond anvils as quantum sensors^22^. Additionally, the screening of applied magnetic fields by the H_3_S superconductor has been explored using the forward nuclear resonant scattering technique, employing the ^119^Sn Mössbauer isotope as a sensor^23^.

The screening of applied magnetic fields, indicating the Meissner state, and the presence of hysteretic loops upon sweeping high applied magnetic fields, evidencing the presence of persistent superconducting currents and a magnetic flux trap, were clearly observed in H_3_S samples in the pioneering work^6^. However, no initial *M*(*H*) data, or so-called virgin curves, were measured, and *H*_*c1*_ and *λ*_*L*_ were only roughly estimated^6^.

Additionally, *M*(*H*) data of H_3_S at *P* = 140 GPa, including the virgin curve, were measured at a single temperature *T* = 100 K, using a magnetic property measurement system (MPMS) from Quantum Design, as reported in ref. ^21^. At that time, we performed conventional cleaning of the diamond anvil cell (DAC) body, diamond anvils, seats, and gaskets to remove possible contamination stemming from the manufacture of these parts using instruments made of steel and tungsten carbide (with impurities of cobalt). However, the conventional cleaning did not completely eliminate the strong paramagnetic contribution of the DAC assembly, which significantly affected the measured total signal. It should also be noted that this DAC had additional elements (electrical leads, wiring, and solder) for electrical transport measurements, which also contributed to the background. Therefore, to minimize the paramagnetic perturbation, we performed these measurements using a standard background subtraction procedure of the MPMS software for the SQUID magnetometer, where the background was collected above *T*_*c*_ at *T* = 210 K (see the inset in Fig. 1).

**Fig. 1:**
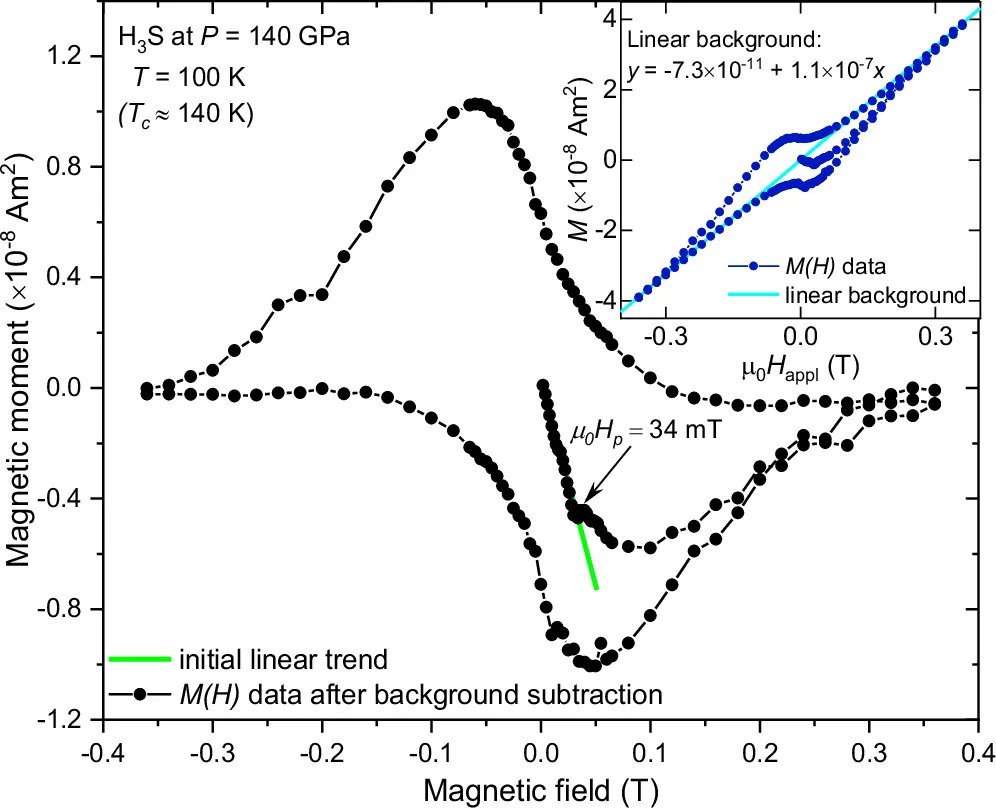
Magnetic moment *M(H)* measurements in H_3_S sample pressurized at *P* = 140 GPa (*T*_*c*_ ≈ 140 K) from ref. ^21^, demonstrating typical superconducting behavior. Raw *M(H)* data, including the background response from the DAC, are shown as blue circles in the inset. In this case, the background is paramagnetic, leading to a positive background slope in the raw data. After the subtraction of the background, approximated by a linear function (sky blue line in the inset), the *M(H)* data exhibits the typical shape for a type-II superconductor. The virgin *M(H)* curve shows a clear deviation from the initial linear trend (green line) at approximately 34 mT.

In our work^1^ we made every effort to avoid all possible sources of contamination in our DACs, which could lead to a significant increase of the paramagnetic background slope in *M(H)* measurements and the automatic background subtraction procedure used in refs. ^6,21^ was not applied for either *M(T)* or *M(H)* magnetic measurements. As a result, the *M(H)* data of DACs with the *Im-3m*-H_3_S and *Fm-3m*-LaH_10_ samples were not distorted by paramagnetic slope from impurities, and showed the diamagnetic slope naturally expected from the magnetic susceptibility values of the materials used for the high-pressure assembly (Fig. 2). More recently, the penetration of magnetic flux into H_3_S and LaH_10_ superconductors was systematically studied using the almost background-independent trapped flux method over a wide temperature range^24^.

**Fig. 2:**
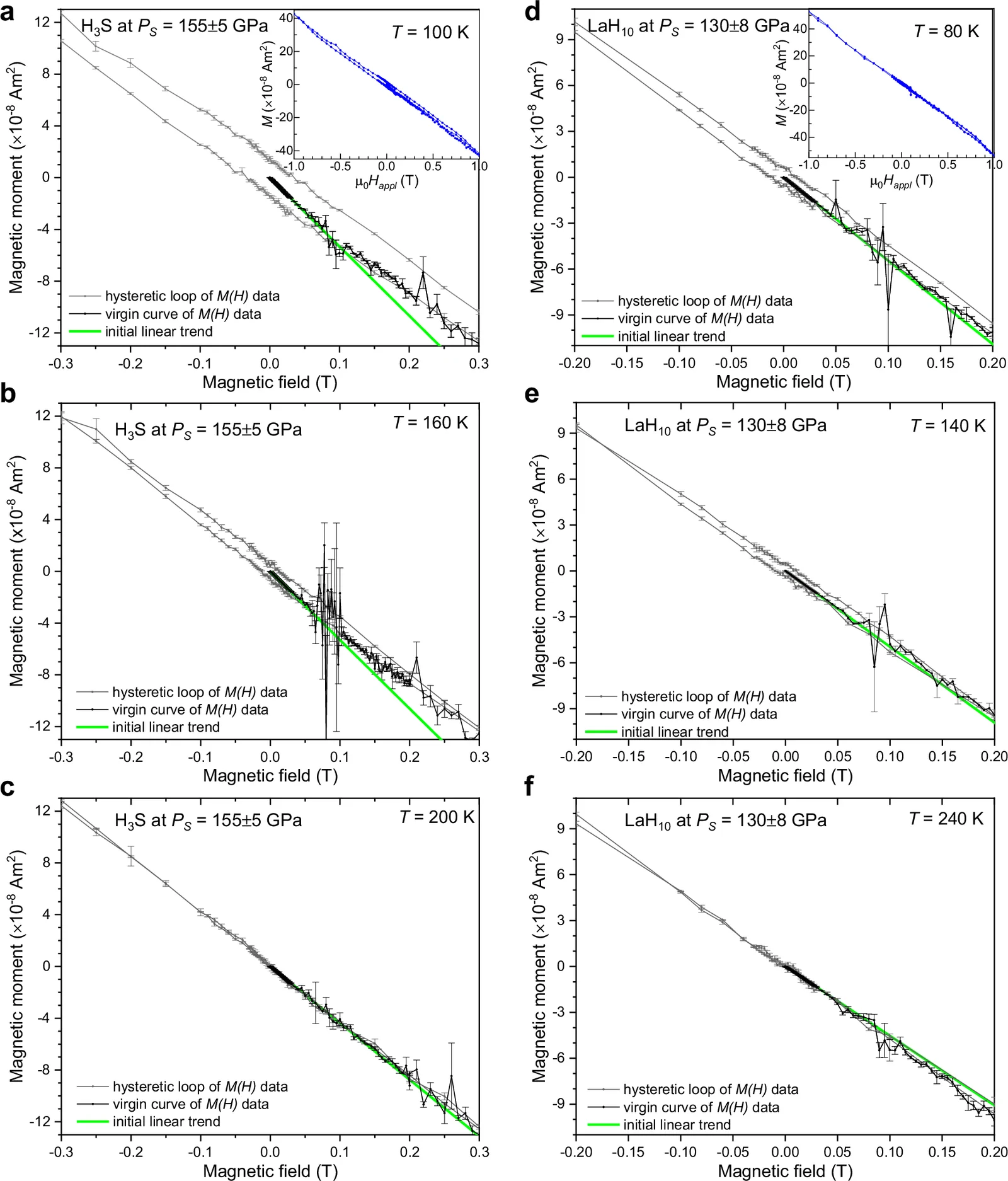
Magnetic moment *M*(*H*) measurements in hydrogen-rich superconductors *Im-3m*-H_3_S and *Fm-3m*-LaH_10_. *M(H)* data upon sweeping applied magnetic fields in the range of −1 to 1 T, including virgin curves, demonstrate: **a**, **b** the presence of characteristic superconducting hysteresis at *T* = 100 K and 160 K (below *T*_*c*_); and **c** the absence of the hysteresis at *T* = 200 K (above *T*_*c*_) in the *Im-3m*-H_3_S sample pressurized at *P*_*S*_ = 155±5 GPa. *M(H)* data upon sweeping applied magnetic fields in the range of −1 to 1 T, including virgin curves, demonstrate: **d**, **e** the presence of characteristic superconducting hysteresis at *T* = 80 K and 140 K (below *T*_*c*_); and **f** the absence of the hysteresis at *T* = 240 K (above *T*_*c*_) in the *Fm-3m*-LaH_10_ sample pressurized at *P*_*S*_ = 130±8 GPa. Black and gray circles correspond to the virgin and hysteretic portion of the *M(H)* data. Green lines represent the initial linear behavior of the virgin *M(H)* data and serve as a guide to demonstrate the deviation of the virgin curve from the linear trend. The measured *M(H)* data showed a diamagnetic background slope for the rigorously etched DAC assembly. Insets demonstrate the whole measured range of *M(H)* datasets for the H_3_S sample at *T* = 100 K and the LaH_10_ sample at *T* = 80 K.

We measured *M(H)* data by sweeping applied magnetic fields in the range of -1 to 1 Tesla (i.e., μ_0_*H*_*appl*_: 0 T → 1 T → 0 T → -1 T → 0 T → 1 T) to obtain virgin curves and hysteretic loops at temperatures above and below *T*_*c*_ of H_3_S and LaH_10_. At each temperature, each *M(H*_appl_*,T)* point was measured 5 or 6 times. Several measured datasets are shown in Fig. 2. Since each *M(H)* loop measurement took several days, at some temperatures in our study, we looked at only the virgin curve (μ_0_*H*_*appl*_: 0 T → 1 T) which is the part of relevance to obtaining *H*_*p*_. In addition, we measured ZFC and FC *M(H*_appl_*,T)* datasets, which are also shown and discussed in ref. ^1^

Below, we describe in detail the procedure for extracting the penetration field *H*_*p*_, the lower critical field *H*_*c1*_, the London penetration depth *λ*_*L*_, and the Ginzburg–Landau parameter *κ* from the experimental data using an alternative approach. Importantly, the choice of procedure does not alter our main experimental result: the hysteresis loops recorded in H_3_S and LaH_10_ (Figs. 1, 2) unambiguously confirm the superconducting state in these highly compressed hydrides.

After accounting for the estimated sample geometry, we obtained μ_0_*H*_*c1*_ values of ≈0.92 T and ≈0.47 T, and *λ*_*L*_(0) of ≈24 nm and ≈36 nm in H_3_S and LaH_10_, respectively. The small values of *λ*_*L*_ indicate a high superfluid density in both hydrides. The corresponding Ginzburg-Landau parameter *κ* ∼13–24 places these compounds in the group of moderate-*κ* type-II superconductors (such as MgB_2_ and A15 alloys), rather than in the category of hard superconductors, as might be intuitively expected given their high *T*_*c*_s. Our later estimated value of *λ*_*L*_(10 K) = 37 nm in H_3_S^24^ does not alter this conclusion.

A key point is how to determine the onset of deviation of the virgin *M*(*H*) part of the data from the linear trend, given noisy data in minor instances. The source of the noise is the S700X SQUID magnetometer, which we used in all our experiments^1^, it utilizes two current sources: the first provides magnetic fields up to 30 mT with low noise, and the second provides higher fields but with higher noise, especially in the field range of 35-150 mT. This design is standard from the manufacturer of the S700X SQUID magnetometer, and we cannot modify it. As a result, we had no problem with the signal-to-noise ratio and the estimation of *H*_*p*_ values when the onset point of deviation from the linear trend lies within the range of magnetic fields <30 mT. Fortunately, this applied magnetic field range covers all measured data for the LaH_10_ sample (and for the majority of well-known superconductors) and partly for the H_3_S sample at *T* = 180 K and 160 K.

The deviation points for the H_3_S sample measured at lower temperatures shift to the range of noisy data. The physical origin for this shift is a large value of the lower critical field, *H*_*c1*_, in H_3_S (or, in other words, short *λ*_*L*_).

Even when the deviation from the linearity is visibly discernible in the original scattered data, the application of smoothing or averaging procedures is required for a more accurate and objective determination of *H*_*p*_. Accordingly, we first applied smoothing to the high-noise regions of the measured *M*(*H*_*appl*_*,T*) datasets, enabling a consistent analysis across the full field range. In retrospect, we accept that the lack of an explicit description of this procedure in our original publication is a valid point of criticism. The procedure we used is indeed open to criticism, because it should have been described at the beginning. We now provide a detailed account of the smoothing, averaging, and filtering procedures employed, as outlined in the accompanying *Author Correction*^25^. Additionally, we present an alternative approach for estimating *H*_*p*_ directly from the raw measured data without any smoothing or filtering, described below.

Since no universally accepted method for determining *H*_*p*_ is established in the literature, its identification is often performed visually by detecting the deviation from the initial linear trend in the virgin *M(H)* curve. Additional uncertainty in type-II superconductors with strong pinning arises from the absence of a sharp change in the virgin *M(H)* curve once *H*_*p*_ is exceeded. This challenge is further amplified in measurements of tiny samples of hydrogen-rich compounds, where the superconducting response is near the sensitivity threshold of the SQUID magnetometer and is perturbed by the magnetic background contribution from the DAC.

We recently developed a generalized method for systematically determining the deviation of a magnetization curve from linear behavior. An identical approach is widely used for the determination of the critical current values in superconductors^3^, and it has been successfully extended to extract *H*_*p*_ from *DC* magnetization data^4^ in various superconductors. This method is also attractive to us because datasets with variable noise levels can be easily analyzed.

The main idea of this method is to fit the measured *M*(*H*_*appl*_) by the power-law function of the applied magnetic field *H*_*appl*_. The power-law function includes a linearly dependent term, which is attributed to the linear Meissner diamagnetic response. The deviation from the linear dependence is described by ${{M}_{c}\times \left(\frac{{H}_{{appl}}}{{H}_{p}}\right)}^{n}$ term, where ${M}_{c}$ is the threshold criterion to define the *H*_*p*_. Thus, ${M}_{c}$ plays a similar role to the electric field criterion of *E*_c_ = 1 μV/cm used to determine the transport critical current density^3^ from the *E*–*J* curves. The details of these methods can be found in refs. ^3,4^. A distinct advantage of this method is that it provides a systematic criterion for detecting *H*_*p*_ values based on phenomenologically established shape of the *M(H)* dependence in the vicinity of *H*_*p*_, whereas previously employed methods rely on a threshold value that varies widely from report to report and are therefore much less consistent. The fitting function is described by Eq. (1):

1 $$M\left({H}_{{appl}}\right)={M}_{0}+k\times {H}_{{appl}}+{{M}_{c}\times \left(\frac{{H}_{{appl}}}{{H}_{p}}\right)}^{n}$$

where *M*_*0*_ is an instrumental offset, *k* is a linear term (also known as the Meissner slope), *M*_*c*_ is the threshold criterion, and *n* is the power-law exponent.

It should be noted that a power-law Eq. (1) is not applicable for fitting the entire *M(H)* curve (similar to the case of *E–J* curves) and must be used solely to find the onset point where the measured magnetic moment starts to deviate from the initial linear trend due to the penetration of magnetic flux into the superconductor. For this reason, one should define *M*_*c*_ and the range of *H*_*appl*_ for reasonable fitting. Identical considerations exist in the determination of the critical current density from the *E*–*J* curves using the power-law approach^3^.

For *M*(*H*) datasets where *H*_*p*_ lies in the low-noise region, we set the *M*_*c*_ criterion as approximately three times the value of the uncertainty of the measured magnetic moment, above which the deviation from the linear trend becomes reliable. We limited the range of *M(H)* data for the fitting to the value of *H*_*appl*_ at which the difference between the measured *M*(*H*_*appl*_) and the Meissner line (linear term) reached approximately ten times the value of *M*_*c*_.

We show several such fits with fitting parameters in Fig. 3 and Supplementary Fig. S1. For better representation of the onset of *H*_*p*_, we subtracted the linear background *y* = *a* + *b* × *x* (*a* and *b* parameters given for each plot). We also reanalyzed the value of *H*_*p*_ for the H_3_S data at *P* ≈ 140 GPa (*T*_*c*_ ≈ 140 K) published in ref. ^21^ The *H*_*p*_ values refined by the new approach are consistent with those estimated in the original works^1,21^.

**Fig. 3:**
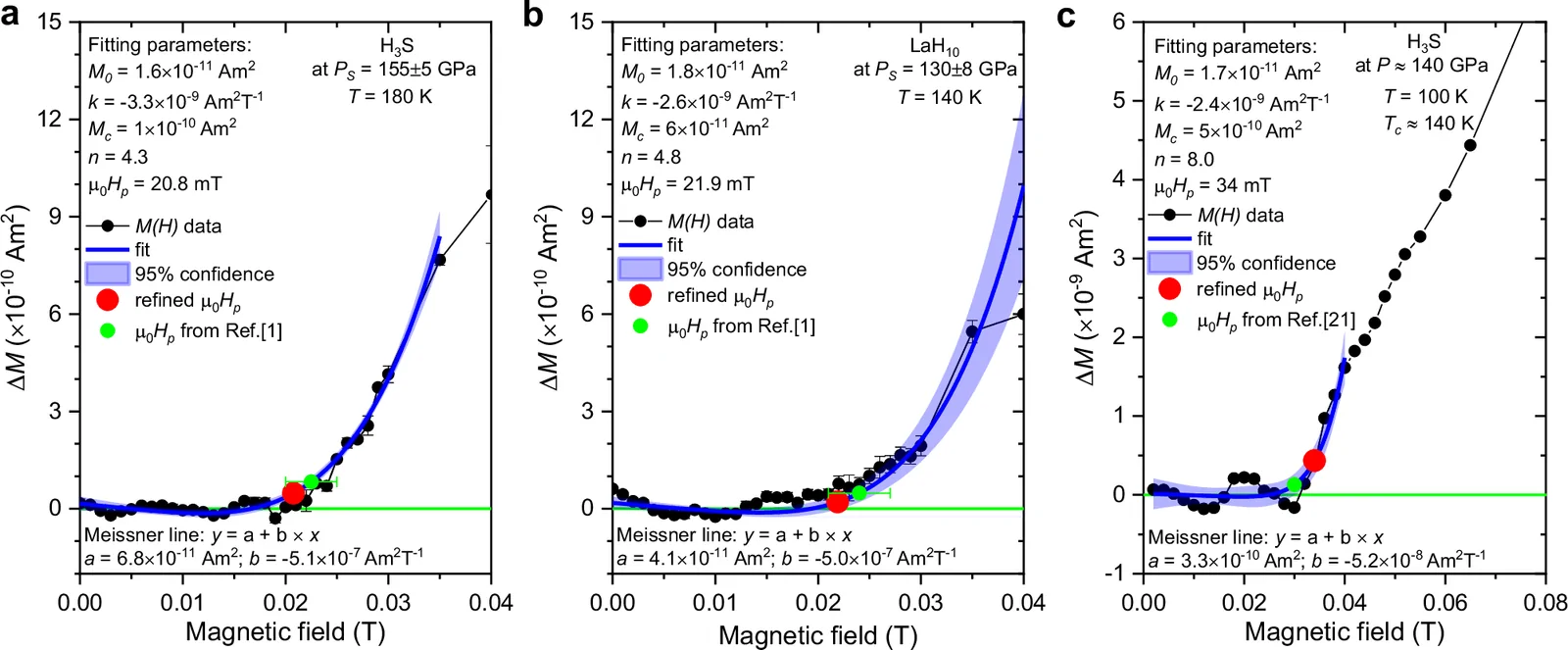
The extraction of μ_0_*H*_*p*_ in the low-noise region of applied magnetic fields (≤ 30 mT). **a** A portion of the *M*(*H*) virgin curve for the *Im-3m*-H_3_S sample at *P*_*S*_ = 155±5 GPa, measured at *T* = 180 K from ref. ^1^; **b** a portion of the *M*(*H*) virgin curve for the *Fm-3m*-LaH_10_ sample at *P*_*S*_ = 130±8 GPa, measured at *T* = 140 K from ref. ^1^; **c** a portion of the *M*(*H*) virgin curve for the H_3_S sample at *P* ≈ 140 GPa (*T*_*c*_ ≈ 140 K), measured at *T* = 100 K from ref. ^21^ We subtracted the linear function of *y* = *a* + *b* × *x* (with *a* and *b* parameters shown below each curve) from the original *M*(*H*) data for better presentation of the initial linear trend. This line represents the Meissner line and is defined as a linear fit of the data measured at low applied magnetic fields below μ_0_*H*_*p*_. Black circles represent the measured *M*(*H*) data; red and green circles represent μ_0_*H*_*p*_ values estimated in the present *Addendum* and in refs. ^1,21^, respectively. The green line depicts the Meissner line (*y* = 0), and blue curves represent the fits with 95% confidence bands.

For *M*(*H*) datasets of the *Im-3m*-H_3_S sample at *P*_*S*_ = 155±5 GPa measured at *T* ≤ 140 K, where μ_0_*H*_*p*_ values lie above 30 mT (in the noisy region), we set the *M*_*c*_ criterion to approximately the averaged value of uncertainties of the *M(H*_*appl*_*)* datasets used for fitting, including both low- and high-noise regions, but not larger than 10% of the maximum diamagnetic response |Δ*M*_*max*_| from the superconducting H_3_S sample. Thus, for the dataset measured at *T* = 140 K, *M*_*c*_ = 8 × 10^-10^ Am^2^, and for the datasets measured at *T* ≤ 120 K, *M*_*c*_ = 1 × 10^-9^ Am^2^. The data range for fitting was limited to magnetic fields at which the difference between the measured *M(H*_*appl*_*)* and the Meissner line (linear term) reached approximately ten times the value of *M*_*c*_, as it was done for the estimation of *H*_*p*_ in the low-noise region. We show these fits with fitting parameters in Fig. 4 and Supplementary Fig. S2. The fitting was performed using data weighting, specifically the “Instrumental” weighting method in Origin software^26^, where each data point is weighted proportionally to $\frac{1}{{\left({error}\right)}^{2}}$.

**Fig. 4:**
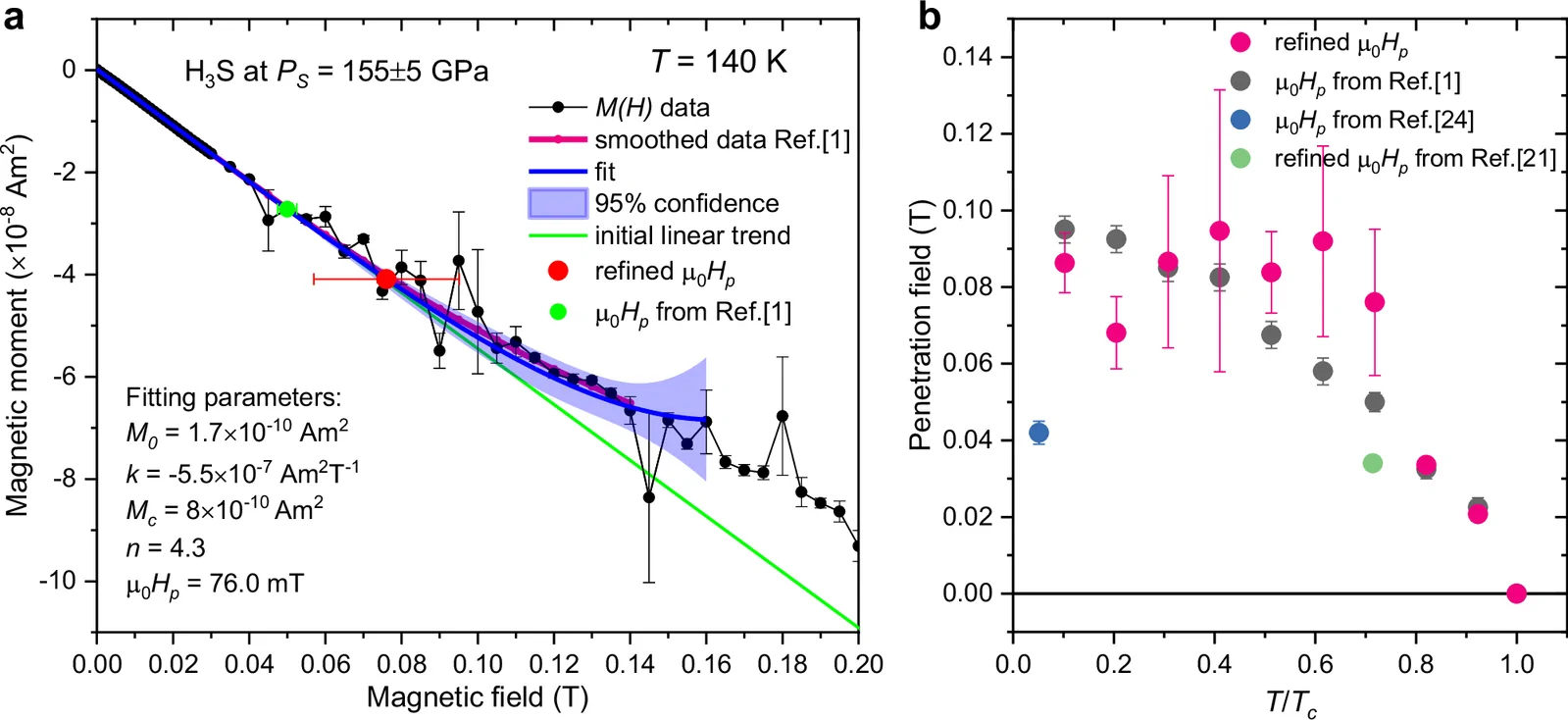
The extraction of μ_0_*H*_*p*_ in the high-noise region of applied magnetic fields ( > 30 mT) for the *Im-3m*-H_3_S sample at *P*_*S*_ = 155±5 G*P*a. **a** Fit of the portion of the *M*(*H*) virgin curve for the *Im-3m*-H_3_S sample at *P*_*S*_ = 155±5 G*P*a, measured at *T* = 140 K by Eq. (1). Fitting parameters are shown on the panel. The smoothed data from ref. ^1^ is shown as a magenta curve. The initial linear trend was defined as a linear fit of the measured data in the applied magnetic fields region of 0–20 mT. **b** Summary plot of the estimated μ_0_*H*_*p*_ values for H_3_S samples measured in different experiments: traditional magnetic susceptibility measurements (magenta circles refined using the present approach, gray circles derived from the smoothed data in ref. ^1^, and green circles from ref. ^21^) and trapped magnetic flux method (blue circle, ref. ^24^).

In traditional magnetization measurements, the total magnetic signal from the pressurized sample unavoidably includes the response of the DAC body. The diamagnetic response of the DAC continuously grows with applied magnetic fields, while the useful signal from the superconducting sample weakens soon after *H*_*appl*_ exceeds *H*_*p*_. This leads to a decrease in the signal-to-background ratio at high applied magnetic fields, but does not affect the estimation of *H*_*p*_ values.

Another important parameter is the background response of DACs to applied magnetic fields. Despite our efforts to manufacture miniature DACs with a background response to applied magnetic fields as low as possible, in practice, the background of the entire high-pressure assembly is not perfectly linear at low applied magnetic fields and across a wide temperature range. Since we cannot significantly increase the size of superconducting samples (the larger diamond anvil culets do not sustain these pressures) to improve the useful signal-to-background ratio, the subtle nonlinearity of the DAC background can manifest in the *M(H)* measurements. However, this feature does not significantly affect the detection of superconductivity in our samples. Several cases for the background dependence of the DAC used in our work^1^ at *T* = 20 K, 180 K, and 200 K are shown in Supplementary Fig. S3. When the sample is in the normal state at 200 K, the virgin *M(H)* curve shows a subtle downturn at approximately 10 mT. The same effect is observed for the data measured at lower temperatures, where μ_0_*H*_*p*_ values exceed 30 mT, i.e., when the nonlinear DAC background interferes with the Meissner state of the superconducting sample (linear magnetic-field dependence of the *M(H*_*appl*_*)*). At sample temperatures at which μ_0_*H*_*p*_ is below 30 mT, the effect of the penetration of the magnetic flux into the superconducting sample surpasses the nonlinearity in the DAC background, allowing for estimation of *H*_*p*_.

We also checked the variation of *H*_*p*_ values upon changing the range of the *M(H)* dataset used for fitting. By extending the *H*_*appl*_ range to two times (μ_0_*H*_*appl*_ = 300 mT) and three times (μ_0_*H*_*appl*_ = 440 mT) for H_3_S sample at *T* = 100 K, the exponent of the fitting function reduces from *n* = 5 to *n* = 2.2, resulting in less accurate fitting of the experimental data (see Supplementary Fig. S4). Despite this, the extracted μ_0_*H*_*p*_ values do not change significantly: from 84±10 mT to 63±9 mT, a difference of approximately 25%. The value of μ_0_*H*_*p*_ reported in ref. ^1^ is 68±4 mT.

Applying the method proposed for the extraction of *H*_p_ from *DC* magnetization measurements in ref. ^4^ to our datasets for the *Im-3m*-H_3_S and *Fm-3m*-LaH_10_ samples reported in ref. ^1^, we obtained the refined values summarized in Fig. 5. In particular, the refined μ_0_*H*_*p*_*(0 K)* = 108±7 mT for the *Im-3m*-H_3_S sample at *P*_*S*_ = 155±5 GPa and 35±3 mT for the *Fm-3m*-LaH_10_ sample at *P*_*S*_ = 130±8 GPa versus 97±2 mT and 39±2 mT for the datasets reported in ref. ^1^

**Fig. 5:**
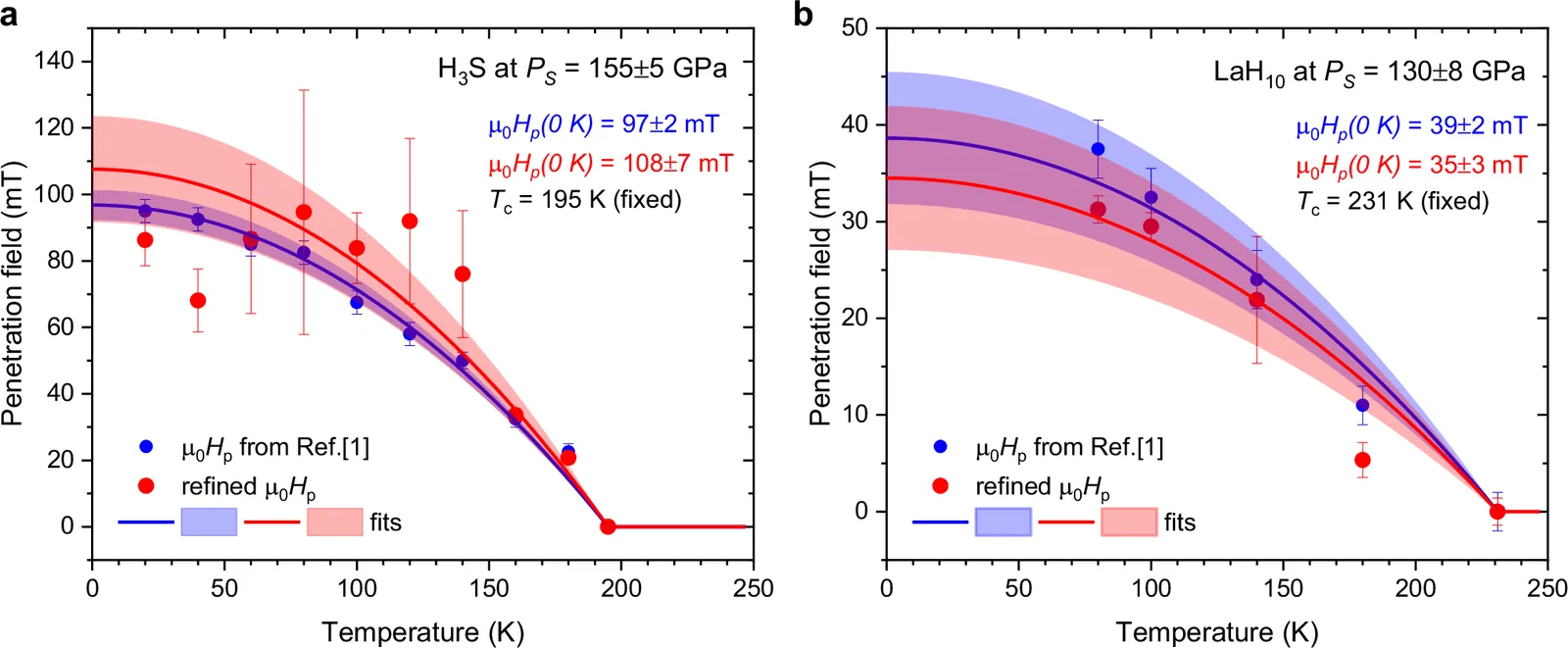
Temperature dependence of estimated μ_0_*H*_*p*_ for the *Im-3m*-H_3_S and *Fm-3m*-LaH_10_ samples. **a** Refined μ_0_*H*_*p*_ values for the *Im-3m*-H_3_S sample at *P*_*S*_ = 155±5 GPa; and **b** refined μ_0_*H*_*p*_ values for the *Fm-3m*-LaH_10_ sample at *P*_*S*_ = 130±8 GPa. Fit of both datasets of estimated μ_0_*H*_*p*_(*T*) values and their extrapolation to μ_0_*H*_*p*_*(0 K)* was done using the equation ${{\mu }_{0}H}_{p}\left(T\right)={{\mu }_{0}H}_{p}(0K)\times \left(1-{\left(\frac{T}{{T}_{c}}\right)}^{2}\right)$. Blue and red circles represent the data from ref. ^1^ and the refined data in the present *Addendum*, respectively. Light blue and light red areas show 95% confidence bands of the fits.

It is noteworthy that estimating *H*_*c1*_*(T)* values necessitates knowing the demagnetizing factor, *N*. We derived the demagnetization correction, (1-*N*)^−1^, from the estimated geometry of the synthesized samples, approximately 8.5 and 13.5 for H_3_S and LaH_10_, respectively. These values assume that the superconducting phases are uniform and shaped as ideal thin solid disks. However, the real sample shape and continuity of the superconducting phase can differ from this ideal case, with the worst scenarios being the sample consisting of separated small crystallites, which could reduce the demagnetization correction (1-*N*)^-1^ factor to the value of 1.5, or the superconducting regions being thinner than the idealised disks, in which case the (1-*N*)^-1^ could be substantially larger than our estimates. The systematic errors on our estimates of *H*_*c1*_*(T)* are therefore substantial, but we believe that the estimate is worth attempting, because *κ* can be obtained as a root of the following Eq. (2)^27,28^:

2 $$\frac{{H}_{c2}}{{H}_{c1}}=\frac{2{\kappa }^{2}}{{{\mathrm{ln}}}\left(\kappa \right)+0.5+\frac{(1+{{\mathrm{ln}}}(2))}{2\kappa -\sqrt{2}+2}}$$

Thus, a valuable estimate of *κ* can be made even in the presence of some uncertainty in *H*_*c1*_, and it is important to know approximately how strongly type-II the hydride superconductors are. The uncertainty in the real value of the demagnetizing factor is not specific to high-pressure measurements but also exists in ambient pressure studies, particularly for polycrystalline samples (e.g., see Fig. 5 in ref. ^29^).

In summary, it is crucial to re-emphasize that we have significantly advanced experimental techniques for studying the properties of novel hydrogen-rich high-temperature superconductors at high pressures using SQUID magnetometry^1,6,21,23,24^. In particular, by utilizing traditional magnetic susceptibility^1,6,21,23^ and new trapped flux^24^ methods, we have demonstrated the presence of all 7 hallmarks of superconductivity in these compounds:

1. Screening and expulsion of the applied magnetic field by the superconducting phase below its *T*_*c*_;
2. The appearance of hysteretic loops by sweeping the applied magnetic field at temperatures below *T*_*c*_, related to persistent superconducting currents in superconductors and their growth with decreasing *T*;
3. The existence of an initial linear trend of *M(H)* in the virgin curve of magnetization data, related to the Meissner state of superconductors.
4. Trapping of magnetic flux by superconductors after switching off the applied magnetic field;
5. Different behaviors in the trapping of magnetic flux under zero-field-cooled and field-cooled protocols arising from the influence of the Meissner state of superconductors.
6. The presence of horizontal temperature-independent plateaus in *M*_*trap*_*(T)* measurements upon reverse cooling superconductors at temperatures below their *T*_*c*_s;
7. Extremely slow creep of the trapped magnetic flux in H_3_S.

In addition, it should be mentioned that the reproducibility and consistency of our results between different magnetic measurements are notable. Both H_3_S and LaH_10_ samples were also measured by the trapped flux method^24^, and the results obtained by the two different techniques agree well with each other. Furthermore, the trapped flux method also provides the estimate of μ_0_*H*_*p*_ (10 K) = 42±3 mT in H_3_S at *P*_*S*_ = 155±5 GPa (diamond anvils in the DAC with LaH_10_ sample were destroyed during measurements), which we consider more accurate since the background of the DAC does not perturb the response of the superconducting sample. The estimations of *H*_*p*_ done by these two methods are consistent. It should be mentioned that in the literature, even ambient pressure superconductors demonstrate much larger dispersion in *H*_*p*_.

In the present *Addendum*, we refined *H*_*p*_*(T)* values for the *Im-3m*-H_3_S and *Fm-3m*-LaH_10_ samples by using an alternative method for determining the deviation from linear behavior in applied magnetic fields in a more consistent manner. The refined *H*_*p*_*(T)* values are commensurate with the previously reported values derived from the smoothed data in ref. ^1^, and fitting curves of the *H*_*p*_*(T)* datasets to the Ginzburg-Landau critical field equation lie within the 95% confidence bands.

Furthermore, we highlight independent confirmations of hydride superconductivity by other groups in the citation list of this *Addendum*, and we note our recent study involving direct probing of the superconducting gap in both H_3_S and D_3_S using electron tunneling spectroscopy^18^, which provides additional evidence of superconductivity in hydrogen-rich compounds under high pressure.

## Supplementary information

Supplementary Information

## Data Availability

The data supporting the findings of this study are openly available at the Open Science Framework (https://osf.io/7wqxb/) with https://doi.org/10.17605/OSF.IO/7WQXB.
