# Author Correction: Magnetic field screening in hydrogen-rich high-temperature superconductors

**DOI:** 10.1038/s41467-026-73704-x

**Published:** 2026-10-06

**Authors:** V. S. Minkov, E. F. Talantsev, V. Ksenofontov, S. L. Bud’ko, F. F. Balakirev, M. I. Eremets

**Affiliations:** 1 Max Planck Institute for Chemistry, Mainz, Germany; 2 M.N. Mikheev Institute of Metal Physics, Ural Branch of the Russian Academy of Sciences, Ekaterinburg, Russian Federation; 3 NANOTECH Centre, Ural Federal University, Ekaterinburg, Russian Federation; 4 Ames Laboratory, U.S. Department of Energy, Iowa State University, Ames, IA USA; 5 Department of Physics and Astronomy, Iowa State University, Ames, IA USA; 6 Los Alamos National Laboratory, Los Alamos, NM USA

**Keywords:** Superconducting properties and materials, Condensed-matter physics

Correction to: *Nature Communications* https://doi.org/10.1038/s41467-022-30782-x, published online 09 June 2022

The original version of the paper^1^ did not provide a sufficiently detailed description of the data-processing steps—specifically, the smoothing, averaging, and filtering procedures—applied to the measured magnetic moment datasets, *M(H*_*appl*_*,T)*, used to determine the penetration field *H*_*p*_. Typically, *H*_*p*_, defined as the field at which magnetic flux begins to penetrate the sample, was determined as the onset of deviation from the linear Meissner response observed in zero-field-cooled (virgin) magnetic moment curves.

In our experiments, we used the S700X SQUID magnetometer, which incorporates two current sources: one provides applied fields up to μ_0_*H*_*appl*_ = 30 mT with low noise, while the other extends the range to higher fields but introduces additional noise, particularly between μ_0_*H*_*appl*_ = 35–150 mT. This design is standard for the instrument and cannot be modified. As a result, measurements below 30 mT exhibit a high signal-to-noise ratio, allowing *H*_*p*_ to be determined with high accuracy whenever the onset of deviation falls within this range. This was the case for all LaH_10_ data (as well as most known superconductors) and partly for the H_3_S at *T* = 180 K and 160 K.

At lower temperatures, however, the deviation points for H_3_S sample shifted into the noisy field range. This behavior arises from the large lower critical field *H*_*c1*_ in H_3_S (or equivalently, its short London penetration depth, *λ*_*L*_).

Because no standard procedure for determining *H*_*p*_ exists in the literature, the onset of deviation is often identified visually. Additional uncertainty in type-II superconductors with strong pinning arises from the absence of a sharp change in the virgin *M(H)* curve once *H*_*p*_ is exceeded. This challenge is amplified in hydrogen-rich samples, where the superconducting signal approaches the sensitivity limits of SQUID magnetometry due to the small sample volume and unavoidable thin-disk geometry (diameter-to-thickness ratio ∼50). In addition, the weak magnetic signal is further perturbed by the background from the diamond anvil cell (DAC).

Even when discernible in the raw scattered data, smoothing or averaging was necessary in the original Article to improve visualization of *H*_*p*_. To address this, we combined low-noise data (μ_0_*H*_*appl*_ ≤ 30 mT) with smoothed data in the noisy range, applying multiple steps of smoothing and averaging where appropriate.

All smoothing, averaging, interpolation, weighting, filtering, and fitting operations were performed using standard tools available in Origin software (version 2021)^2^. The implemented smoothing methods allow for variations in both window size (number of merged data points) and dataset length selected for processing, parameters that can influence the final result. Additionally, because the noise in different datasets originates from random sources, individual outlier points (independently measured scans) can significantly deviate from interpolated values in low-noise regions.

The exact sequence of smoothing/averaging/interpolation operations used for each *H*_*p*_ value in Figure 3 of the original Article was not recorded, as no universal procedure was applicable. Each dataset was treated individually depending on its specific noise structure. Nevertheless, the general approach is outlined below, with examples provided for datasets shown in Figure 3 in ref. ^1^:

1. *Data collection and averaging*: Each data point was measured 5–6 times independently. For each applied magnetic field, the mean value and standard deviation were calculated.
2. *Outlier rejection*: Outliers deviating by more than ∼3*σ* were excluded to minimize artefacts from random SQUID power-supply spikes.
3. *Hysteresis-loop support*: Full hysteresis-loop data, which exhibited lower noise, were used as a reference to identify outliers in the virgin curve where necessary.
4. *Smoothing*: Standard Origin smoothing functions (*e.g*., adjacent averaging, FFT filtering) were applied depending on the local noise level and behavior of the dataset.
5. *Scope limitation*: Only high-noise regions (typically μ_0_*H*_*appl*_ > 35 mT) were smoothed. The low-noise region (0–30 mT), where *H*_*p*_ values were determined for approximately half of the cases, was not treated.

## Example application of the procedure to noisy datasets

- *M(H)* data for *Im-3m*-H_3_S at 100 K
  For this set of measurements, we used the original dataset, specifically the averaged values of *M(H)* obtained from five independent scans at each applied field. The linear background was defined as:
  $$y=\,-5.23\times {10}^{-11}-\left(4.06\times {10}^{-7}\right)\times x$$
  using a linear fit to the hysteresis-loop dataset without a weighting scheme (green line in Figure 1). This background was subtracted from the measured *M(H)* data to remove the main diamagnetic contribution from the DAC body, as illustrated in Figure 1.
  We then applied one of the smoothing methods available in the Origin software to reduce noise in the high-noise portion of the dataset. Specifically, an FFT filter with a 9-point window and no weighting scheme was used. The low-noise virgin portion of the dataset (0 ≤ μ_0_*H*_*appl*_ ≤ 30 mT) was subsequently retrieved and combined with the smoothed data. The result of this procedure is shown in Figure 2, where the smoothed virgin *M(H)* curve provides a clearer demonstration of the onset of deviation from the linear trend.
  Next, we reinstated the previously subtracted linear background to compare the *M(H)* data before and after smoothing. A magnified view of the unprocessed and smoothed *M(H)* datasets (from −0.1 to +0.1 Tesla) is presented in Figure 3, illustrating the effectiveness of the smoothing procedure.
  Finally, we estimated the penetration field *H*_*p*_ using the smoothed virgin *M(H)* dataset with the linear background removed (Figure 2b). In Figure 4, the deviation of the virgin curve from the linear trend (blue line) is clearly visible. From this analysis, we estimate μ_0_*H*_*p*_ ≈ 82 mT, which is in good agreement (considering the noise level) with both the estimate of μ_0_*H*_*p*_ ≈ 67.5 mT reported in the original paper^1^ and the re-evaluated value of μ_0_*H*_*p*_ = 84 ± 11 mT obtained using a newly introduced method (see *Addendum*)^1^.
- *M(H)* data for *Im-3m*-H_3_S at 180 K
  Although the estimated *H*_*p*_ at *T* = 180 K lies within the low-noise region of the *M(H)* data, we nevertheless applied a smoothing procedure to the high-noise portion above 30 mT. Figure 5 shows the measured *M(H)* dataset for the *Im-3m*-H_3_S sample at 180 K. As in the 100 K case, each data point corresponds to the average of five independent scans at the same applied field.
  The zoomed-in region (from −0.02 to 0.12 Tesla) is presented in the right panel of Figure 5. For clarity, all five independent scans of the virgin curve are shown in distinct colors (green, blue, navy, dark yellow, purple). Several scans deviate significantly from the hysteresis loop, which has much smaller error bars (standard deviations). These deviations result from sudden flux jumps caused by SQUID magnet performance in the 35–150 mT field range.
  Some scans returned *M(H)* = 0 Am^2^ due to failed signal detection; these “bad scans” (marked as “0” in Figure 5b) were excluded from averaging. Outliers were defined as scans exceeding *3σ* of the hysteresis-loop data, specifically:
  **Scan 1:** 85, 90, 95, 100 mT
  **Scan 2**: 95 mT
  **Scan 3**: 80, 85, 90, 95 mT
  **Scan 4**: 60, 90, 100 mT
  **Scan 5:** 55, 65, 85 mT
  The linear background (green line) was defined using a linear fit to the hysteresis-loop data (unweighted):
  $$y=\,-2.48\times {10}^{-11}-\left(4.30\times {10}^{-7}\right)\times x.$$
  After subtracting this background, we applied two smoothing steps: adjacent averaging (AAv, 13-point window) and FFT filtering (9-point window), both unweighted, as implemented in Origin. The result is shown in Figure 6a. The low-noise portion of the virgin curve (0–30 mT) was then restored. The onset of deviation from the initial linear trend (blue line) occurs at μ_0_*H*_*p*_ ≈ 23 mT, in good agreement with the value μ_0_*H*_*p*_ ≈ 22.5 mT reported in the original paper^1^ and re-evaluated value of μ_0_*H*_*p*_ ≈ 20.8 mT (Figure 6b, see also *Addendum*)^1^.
- *M(H)* data for *Im-3m*-H_3_S at 160 K
  The estimated *H*_*p*_ value at *T* = 160 K also falls within the low-noise region, but we again applied smoothing to the high-noise portion above 30 mT. Figure 7 shows the *M(H)* data, where each point of the virgin curve represents the average of five independent scans, and the hysteresis-loop data represent the average of six scans. The linear background was defined as:
  $$y=\,-5.11\times {10}^{-11}-\left(4.24\times {10}^{-7}\right)\times x.$$
  Outliers exceeding *3σ* relative to the hysteresis-loop data were excluded. These correspond to the following applied fields:
  **Scan 1**: 60, 65, 67.5, 70, 72.5, 80, 95, 97.5 mT
  **Scan 2**: 65, 67.5, 75, 77.5, 82.5, 85, 87.5, 90, 95, 97.5, 100 mT
  **Scan 3**: 77.5, 80, 82.5, 87.5, 92.5, 95, 97.5, 100, 102.5 mT
  **Scan 4**: 45, 57.5, 70, 72.5, 75, 77.5, 80, 82.5, 85, 90, 102.5 mT
  **Scan 5**: 60, 67.5, 75, 77.5, 92.5, 95, 97.5, 100, 102.5 mT
  These excluded points are highlighted in Figure 7b.
  After subtracting the linear background, we applied two smoothing steps: adjacent averaging (AAv, 5-point window) and FFT filtering (9-point window), both unweighted. The low-noise portion of the dataset (0–30 mT) was then restored. The result is shown in Figure 8. The onset of deviation from the initial linear trend (blue line) is observed at μ_0_*H*_*p*_ ≈ 32 mT, in excellent agreement with both the originally reported value (μ_0_*H*_*p*_ ≈ 32.5 mT) and the re-evaluated (μ_0_*H*_*p*_ ≈ 33.9 mT) (see *Addendum*)^1^.
- *M(H)* data for *Im-3m*-H_3_S at 140 K
  Following the same procedure, the linear background for the *M(H)* dataset measured at *T* = 140 K was defined as:
  $$y=\,-2.87\times {10}^{-9}-\left(4.22\times {10}^{-7}\right)\times x.$$
  The following outliers were excluded:
  **Scan 1:** 100, 220 mT
  **Scan 2:** 80, 100, 145 mT
  **Scan 3**: 90, 180, 200, 220 mT
  **Scan 4**: 95, 220 mT
  **Scan 5**: 45, 90, 95, 105, 110, 160, 180, 200 mT
  We then applied FFT smoothing (9-point window) (see Figure 9) and subsequently restored the low-noise portion of the data (0 ≤ μ_0_*H*_*appl*_ ≤ 30 mT). The resulting dataset (Figure 10) shows a deviation from the initial linear trend at μ_0_*H*_*p*_ ≈ 50 mT, consisting of both the originally reported value (μ_0_*H*_*p*_ ≈ 50 mT) and the re-evaluated value (μ_0_*H*_*p*_ ≈ 76 ± 20 mT).
- *M(H)* data for *Im-3m*-H_3_S at 120 K
  At this temperature, only the virgin curve was measured for the *Im-3m*-H_3_S sample. The linear background (green line) was defined between two endpoints of the *M(H)* dataset, namely at 0 and 1 Tesla:
  $$y=\,-7.98\times {10}^{-11}-\left(4.42\times {10}^{-7}\right)\times x.$$
  The following outliers were excluded:
  **Scan 1**: 70, 95, 100, 105 mT
  **Scan 2**: 60, 70, 75, 90, 110 mT
  **Scan 3**: 60, 75, 80, 85, 90, 95, 110, 145, 200, 210 mT
  **Scan 4**: 65, 75, 80, 85, 90, 95, 105, 140 mT
  **Scan 5**: 55, 75, 85, 90, 100 mT
  We then applied two successive smoothing procedures—adjacent averaging (7-point window) followed by FFT filtering (5-point window)—and subsequently restored the low-noise portion of the data. The resulting smoothed curve (see Figure 11) shows a deviation from the initial linear trend at μ_0_*H*_*p*_ ≈ 60 mT, consistent with the originally reported value (μ_0_*H*_*p*_ ≈ 57.5 mT) and the re-evaluated value (μ_0_*H*_*p*_ ≈ 92 ± 25 mT).
- *M(H)* data for *Im-3m*-H_3_S at 40 K
  At *T* = 40 K, only the virgin-curve dataset was measured. The linear background (green line) was defined as:
  $$y=\,-2.13\times {10}^{-10}-\left(3.04\times {10}^{-7}\right)\times x.$$
  The following outliers were excluded from averaging:
  **Scan 1**: 80, 85, 95, 110, 115, 210 mT
  **Scan 2:** 65, 90, 95, 110, 140 mT
  **Scan 3**: 50, 80, 90, 95, 110 mT
  **Scan 4**: 50, 75, 80, 85, 100 mT
  **Scan 5**: 50, 85, 90, 95, 100, 210 mT
  We then applied adjacent averaging (5-point window) followed by FFT filtering (5-point window) to the cleaned dataset (see Figure 12). After subsequently restoring the low-noise region (0 ≤ μ_0_*H*_*appl*_ ≤ 30 mT), the resulting smoothed curve (see Figure 13) shows the onset of deviation from the linear trend at μ_0_*H*_*p*_ ≈ 85 mT. This is in good agreement with both the originally reported value (μ_0_*H*_*p*_ ≈ 92 mT) and the re-evaluated value (μ_0_*H*_*p*_ ≈ 68 ± 10 mT).
- *Summary plot of virgin-curve deviation at different temperatures*
  Finally, we explain how the composite plot (see Figure 14) demonstrates the deviation of the virgin *M(H)* curves from the linear trend at different temperatures. In Figure 14a, we show five smoothed and background-subtracted *M(H)* datasets measured below *T*_*c*_ ≈ 196 K. For clearer visualization, we further subtracted linear functions from datasets measured at 160 K, 140 K, and 120 K (as specified in Figure 14b). This normalization aligned all curves to a common initial linear trend, enabling a direct comparison of *H*_*p*_ values across temperatures.
- *M(H)* data for *Fm-3m*-LaH_10_
  We now turn to the second set of *M(H)* data, measured for the LaH_10_ sample. From the beginning, it was clear that the magnetic background from the diamond anvil cell used for LaH_10_ sample was larger than that used for H_3_S, as shown in Figures 2a and 2b of the original paper^1^. At that time, this increased background made it more challenging to visualize the onset of deviation from the linear trend in the virgin *M(H)* curve. For this reason, we subtracted the *M(H)* data measured at *T* = 240 K (i.e., above *T*_*c*_ ≈ 230 K, where the sample is in the normal metallic state) from the data measured at temperatures below *T*_*c*_.
  In Figure 15, we present two original *M(H)* datasets measured at 240 K and 80 K. At 240 K – well above the superconducting transition – no hysteresis loop indicative of superconductivity is observed. In contrast, at 80 K (Figure 15b), significantly below *T*_*c*_, a clear hysteresis loop is visible. For both cases, we also show the five independent scans underlying each averaged point of the virgin curve. It is apparent that some individual scans deviate strongly from the maximum expected magnetic moment, as defined by the hysteresis loop obtained from sweeping the magnetic field over a wide range (from -1 to +1 Tesla). Some scan points even fall outside the plotted range. These outlier scans contributed to the averaged values (red circles) and were treated as outliers.
  Figure 15c shows the direct subtraction, *M(H, T* = 80 K)–*M(H, T* = 240 K), performed without re-averaging or excluding outliers, alongside the smoothed final curve from Figure 3d of our original paper^1^ (green curve). The smoothed curve closely follows the overall behavior of the raw subtraction result, even without prior preprocessing of the datasets. While both datasets involved in the subtraction contain partially high-noise regions, the final difference curve remains sensitive to outliers, especially in the noisy interval (30 ≤ μ_0_*H*_*appl*_ ≤ 150 mT). Nevertheless, the green curve agrees well with the direct subtraction, even without removing these outliers.
  It is important to emphasize that subtracting the *M(H)* data measured in the normal state at 240 K from those measured below *T*_*c*_ is not strictly necessary to detect the onset of deviation from linearity. This deviation occurs in the low-field region, prior to the high-noise interval above μ_0_*H*_*appl*_ = 30 mT. Therefore, the subtraction used in the original paper^1^ was somewhat excessive, serving mainly as a visual aid to guide the reader through the scattered high-field data.
  More importantly, the presentation style of the *M(H)* data for the *Fm-3m*-LaH_10_ sample does not significantly affect the estimation of *H*_*p*_. The *H*_*p*_ values reported in the original paper^1^ and the re-evaluated values estimated in the *Addendum*^1^ – now using a mathematically grounded fitting procedure—are in good agreement.

In addition, we developed a generalized, smoothing-free method for systematically detecting deviations from linearity in virgin *M(H)* curves. In this approach, the measured data are fitted with a power-law function of the applied field. The linear term represents the Meissner response, while the second power-low exponent term describes the deviation near *H*_*p*_. This method provides a consistent, criterion-based determination of *H*_*p*_, in contrast to threshold-based methods that vary widely across reports.

Our analysis shows that the *H*_*p*_ values remain stable across different smoothing methods and are in excellent agreement (within uncertainties) with those obtained using this systematic method. Notably, the uncertainty in the demagnetization factor *N* (used to estimate *H*_*c1*_) – which in our samples can be as high as ∼10—exceeds the uncertainty in *H*_*p*_ determination by any of the applied methods.

Details of the new method and the re-evaluated *H*_*p*_ values for H_3_S and LaH_10_ samples are summarized in the *Addendum*^1^. Importantly, the revised *H*_*p*_*(T)* values remain consistent with those reported in the original version of the paper^1^, and the fits of *H*_*p*_*(T)* to the Ginzburg-Landau equation lie within the 95% confidence bands.

**Fig. 1:**
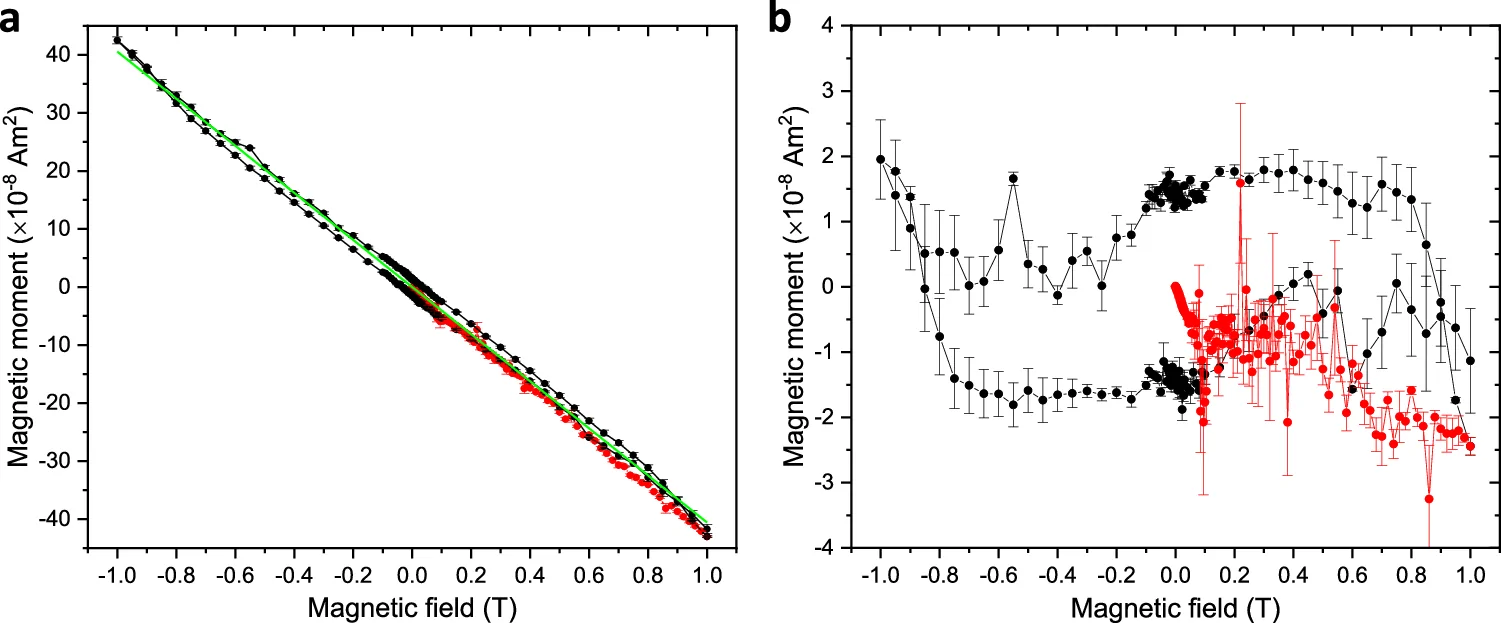
Subtraction of a linear background from the measured *M(H)* dataset for *Im-3m*-H_3_S at *T* = 100 K. **a** Original measured dataset, where each point represents the average of five independent scans; error bars denote the standard deviations. The green line indicates the defined linear background. **b** *M(H)* data after subtraction of the linear background. For clarity, the virgin magnetization curve is shown in red, and the hysteretic loop is shown in black.

**Fig. 2:**
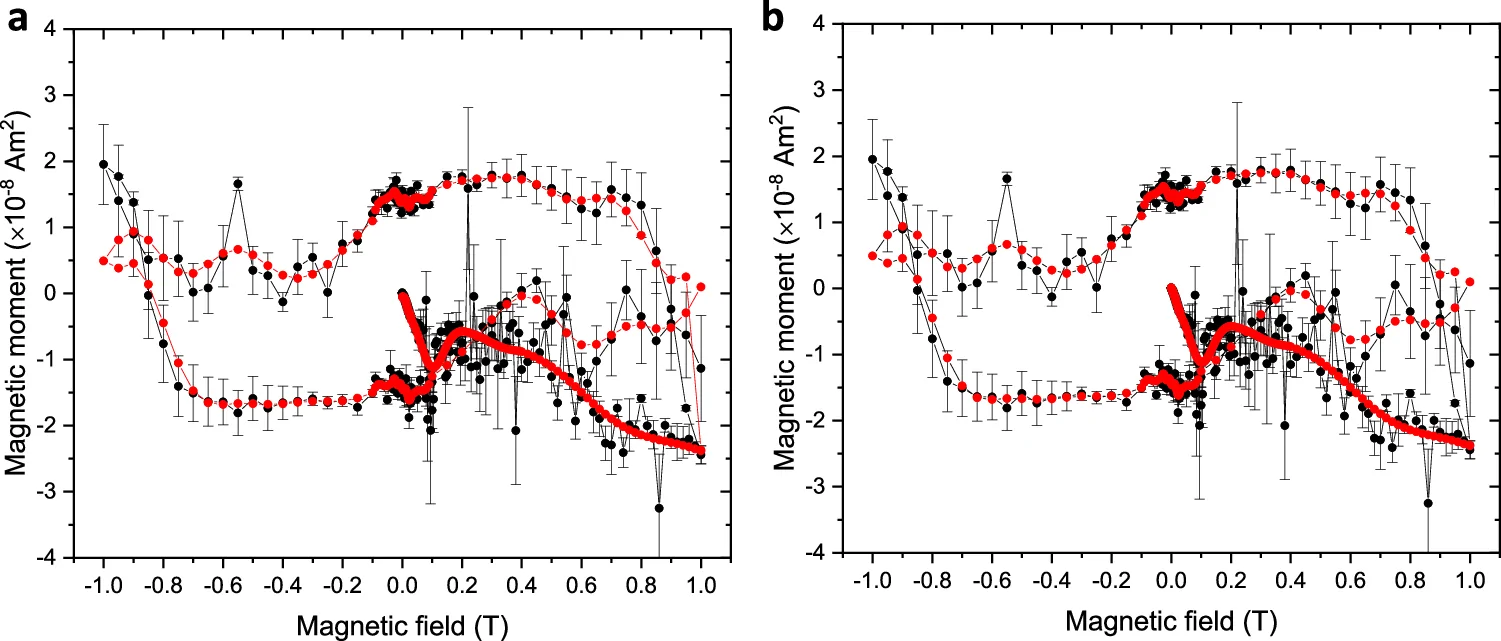
Smoothing of the *M(H)* dataset for *Im-3m*-H_3_S at *T* = 100 K and retrieving the low-noise portion of the virgin curve. **a** *M(H)* data after smoothing with an FFT filter using a 9-point window and no weighting scheme. **b** *M(H)* data after the low-noise portion of the virgin curve (0 ≤ μ_0_*H*_*appl*_ ≤ 30 mT) was retrieved and combined with the smoothed data. In both panels, the original measured hysteretic loop data, with each point representing the average of five independent scans, are shown in black. In panel a, the smoothed M(H) data are shown in red. In panel b, the combined M(H) data, obtained by merging the raw data in the low-noise region with the smoothed data in the noisy region, are shown in red.

**Fig. 3:**
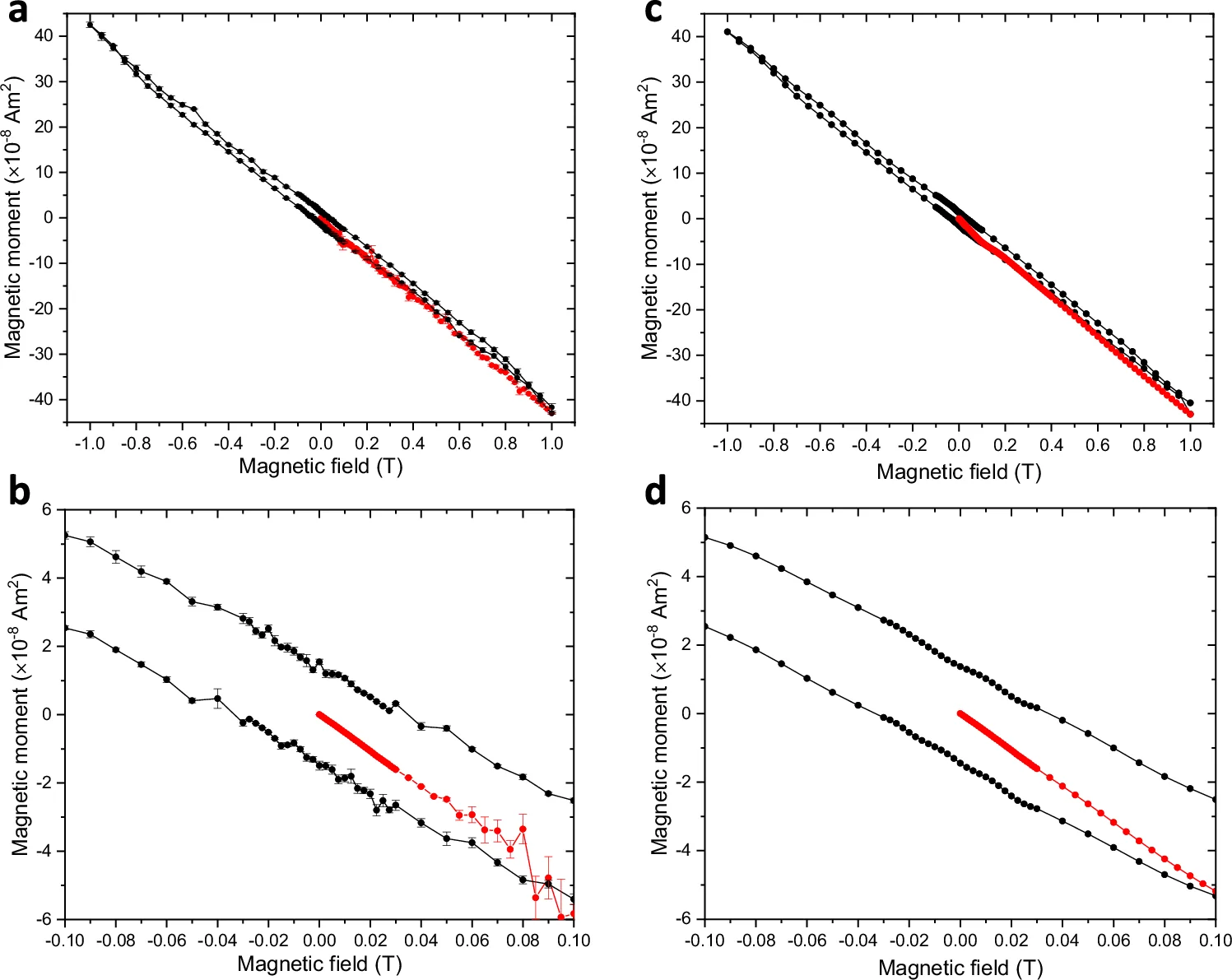
Comparison of the original and smoothed *M(H)* datasets for the *Im-3m*-H_3_S sample measured at 100 K. **a** Original measured *M(H)* dataset. **c** Smoothed *M(H)* dataset for panel **a**. **b** Unprocessed *M(H)* dataset in the range −100 ≤ μ_0_*H*_*appl*_ ≤ 100 mT, near μ_0_*H*_*p*_. **d** Corresponding smoothed dataset in the same range as for panel b.

**Fig. 4:**
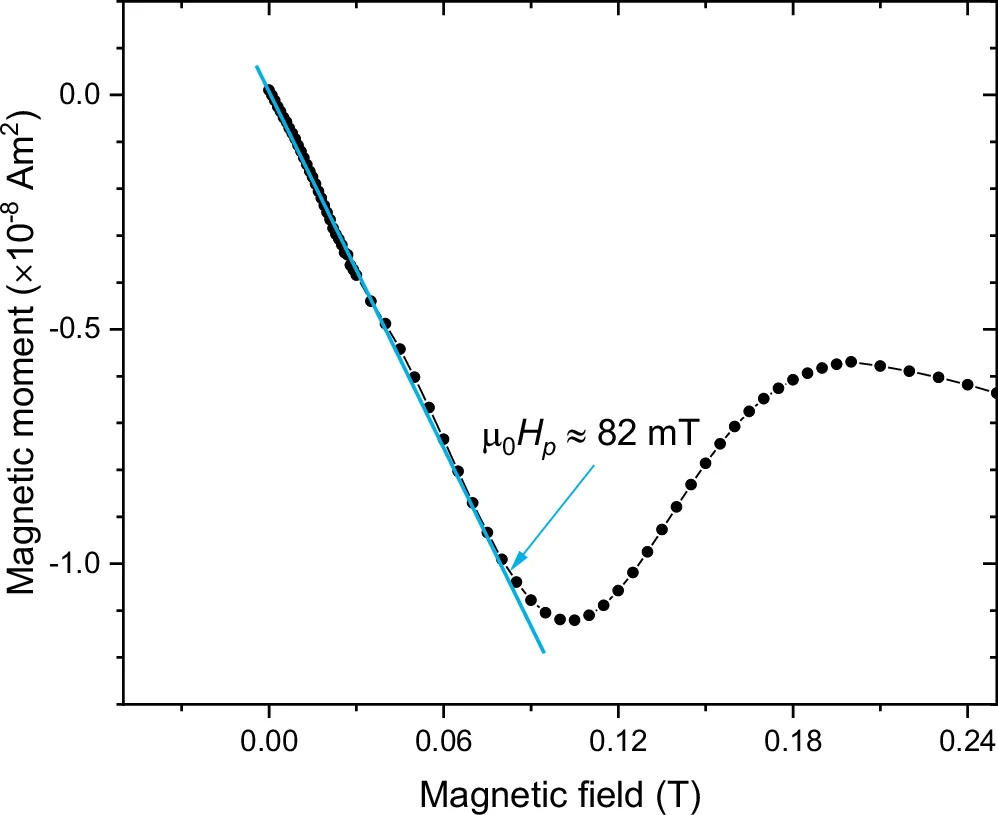
Estimation of the μ_0_*H*_*p*_ value in H_3_S at 100 K using the smoothed virgin portion of the *M(H)* dataset after subtraction of the linear background. The blue line represents the initial linear behavior.

**Fig. 5:**
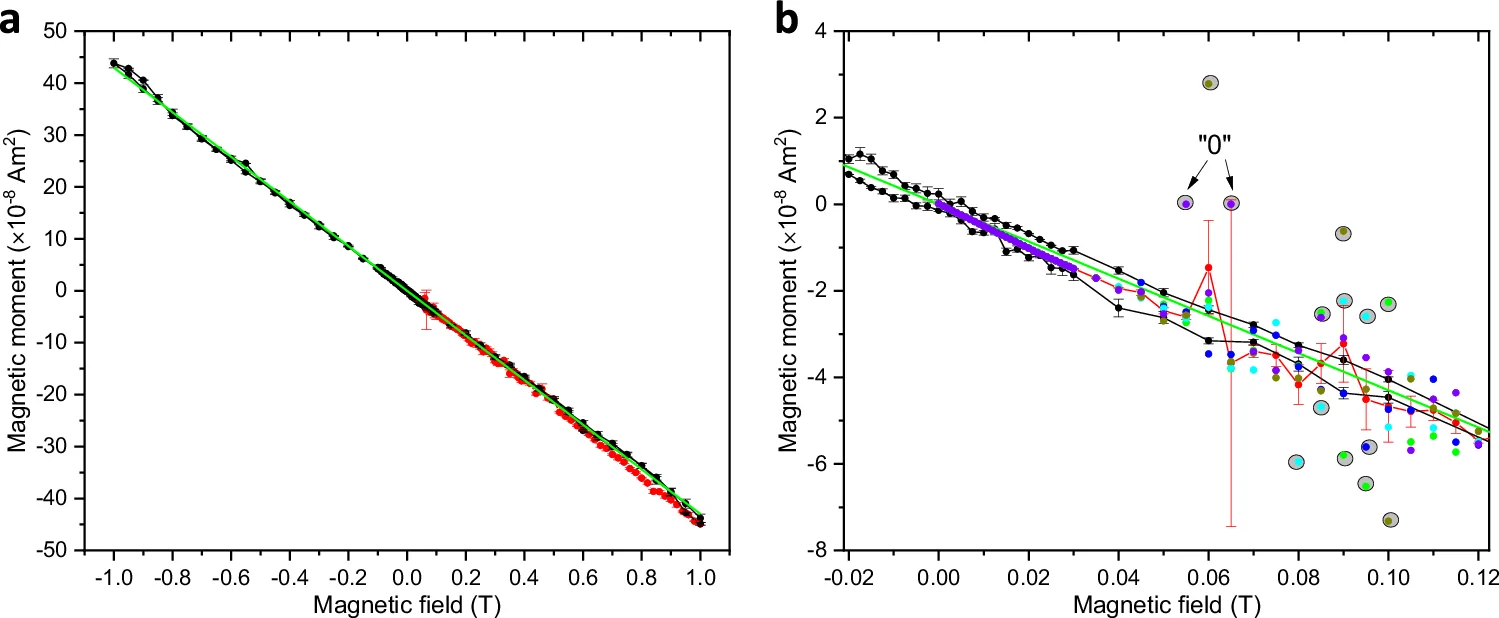
Subtraction of a linear background from the measured *M(H)* data for *Im-3m*-H_3_S at *T* = 180 K and omission of outliers from the high-noise region of the virgin curve. **a** Original measured dataset, where each point represents the average of five independent scans; error bars indicate the standard deviation. The green line shows the defined linear background. **b** Virgin-curve dataset with the outlying points highlighted in the high-noise region. The red and black symbols denote the virgin curve and hysteresis-loop datasets, respectively. Gray circles mark the excluded outliers identified from the five independent scans (shown as blue, navy, green, purple, and dark yellow circles).

**Fig. 6:**
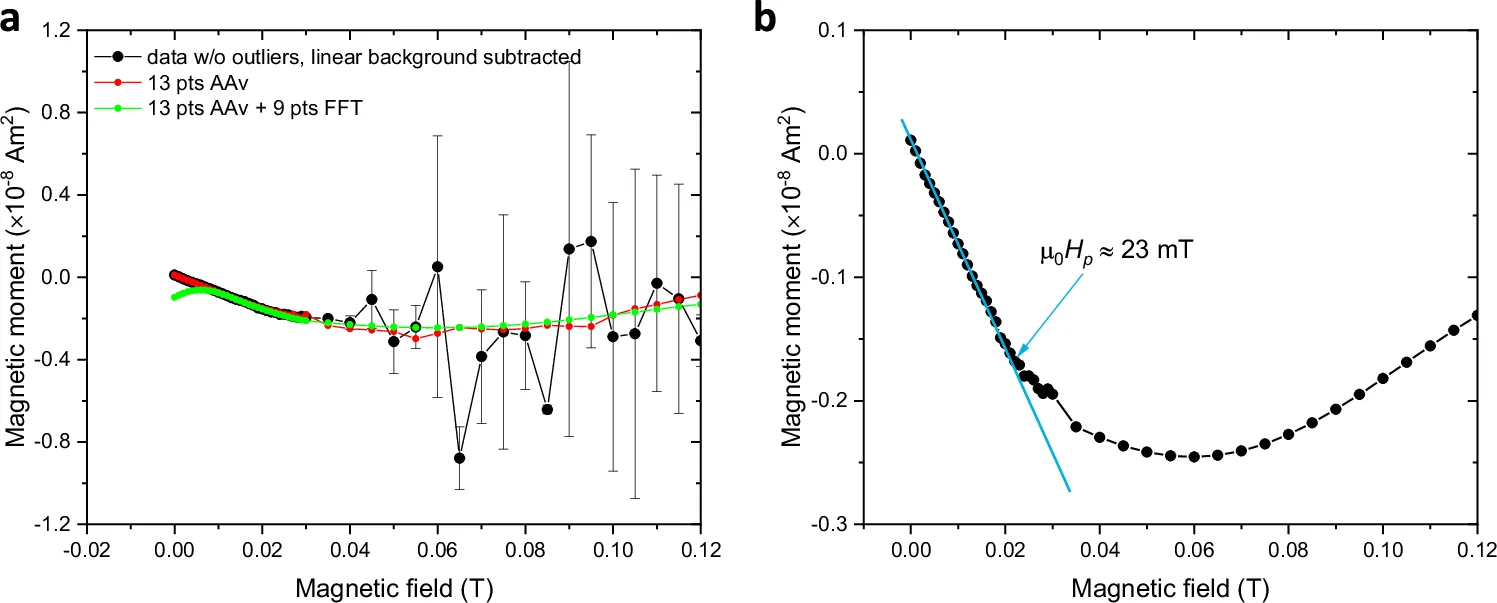
Smoothing of the high-noise portion of the virgin *M(H)* dataset and estimation of μ_0_*H*_*p*_ value in H_3_S at *T* = 180 K. **a** Smoothed *M(H)* dataset after subtraction of the linear background and exclusion of outliers in the high-noise region low-field deviation of the 13 pts AAv + 9 pts FFT curve from the measured data is an artifact of the Smoothing procedure. **b** Estimation of the μ_0_*H*_*p*_ value from the smoothed virgin curve. The blue line represents the initial linear behavior.

**Fig. 7:**
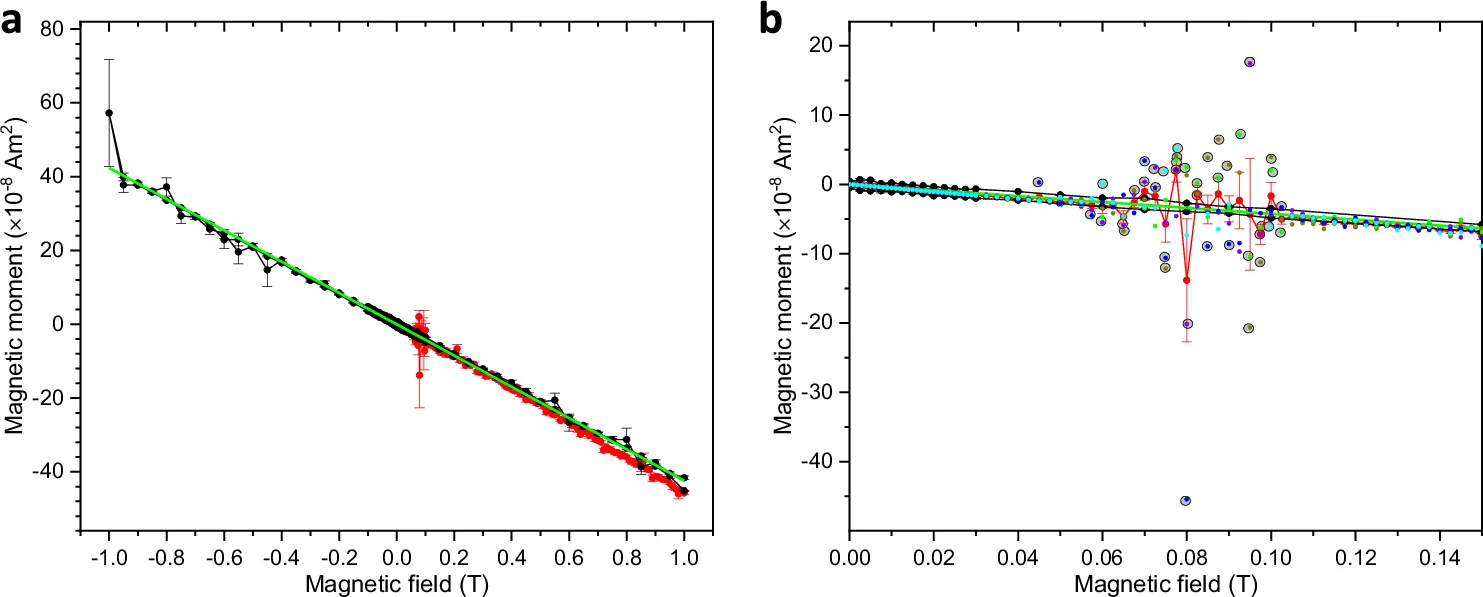
Subtraction of a linear background from the measured *M(H)* data for *Im-3m*-H_3_S at *T* = 160 K and identification of outliers in the high-noise region of the virgin curve. **a** Original measured dataset, where each point represents the average of five independent scans in the virgin curve and six scans in the hysteresis loop; error bars indicate the standard deviations. The green line shows the defined linear background. **b** Virgin-curve dataset with the outlying points highlighted in the high-noise region. The red and black symbols denote the virgin curve and hysteresis-loop datasets, respectively. Gray circles indicate the excluded outliers identified from the five independent scans (shown as blue, navy, green, purple and dark yellow circles).

**Fig. 8:**
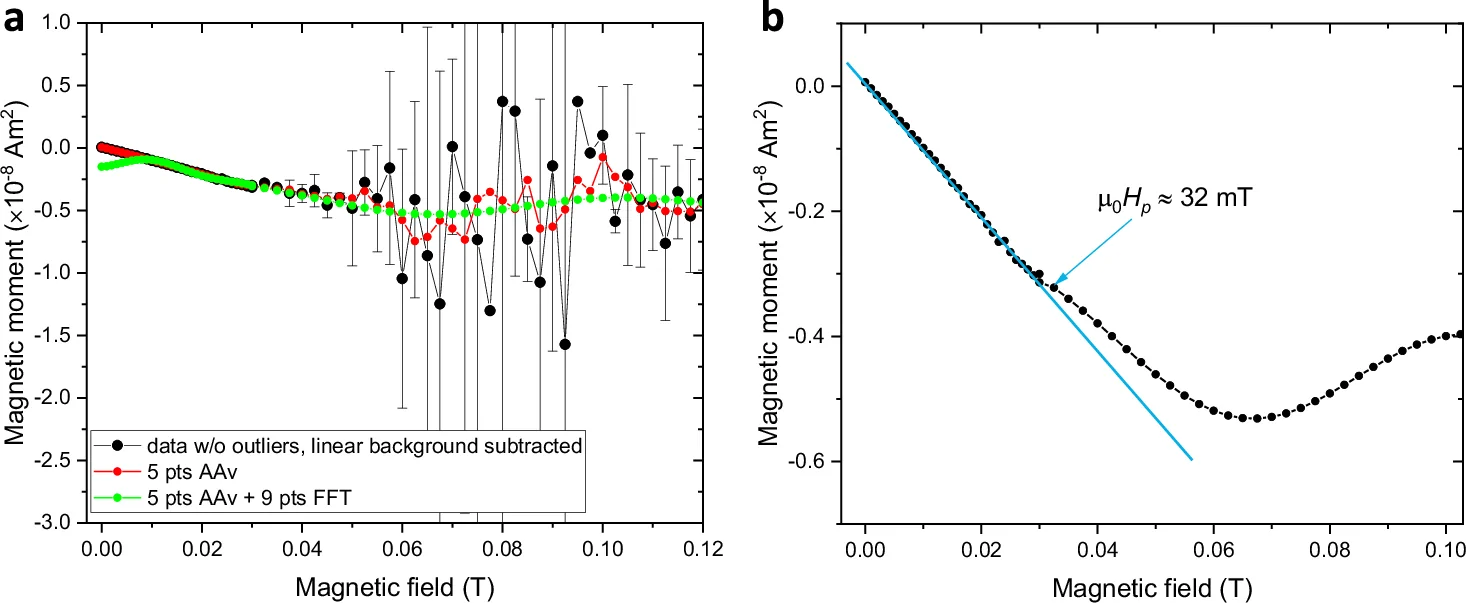
Smoothing the high-noise portion of the virgin *M(H)* dataset for *Im-3m*-H_3_S and estimation of μ_0_*H*_*p*_ value at *T* = 160 K. **a** Smoothed *M(H)* dataset after subtraction of the linear background and exclusion of outliers in the high-noise region low-field deviation of the 5 pts AAv + 9 pts FFT curve from the measured data is an artifact of the Smoothing procedure. **b** Estimation of the μ_0_*H*_*p*_ value from the smoothed virgin curve. The blue line represents the initial linear behavior.

**Fig. 9:**
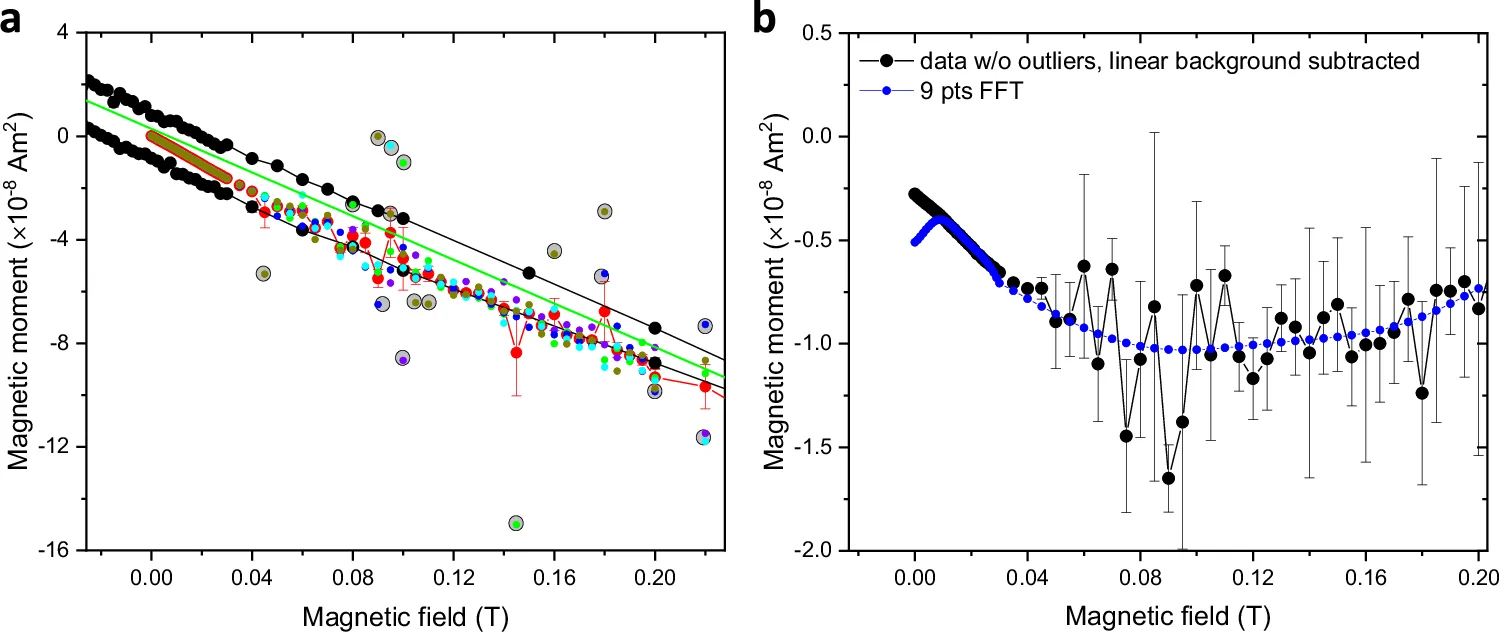
Subtraction of a linear background from the measured *M(H)* data for *Im-3m*-H_3_S at *T* = 140 K and omission of outliers from the high-noise region of the virgin curve. **a** Original measured dataset, where each point represents the average of five independent scans; error bars indicate the standard deviation. Virgin-curve dataset with the outlying points highlighted in the high-noise region. The red and black symbols denote the virgin curve and hysteresis-loop datasets, respectively. The green line shows the defined linear background. Gray circles mark the excluded outliers identified from the five independent scans (shown as blue, navy, green, purple, and dark yellow circles). **b** Smoothed *M(H)* dataset after subtraction of the linear background and exclusion of outliers in the high-noise region low-field deviation of the 9 pts FFT curve from the measured data is an artifact of the Smoothing procedure.

**Fig. 10:**
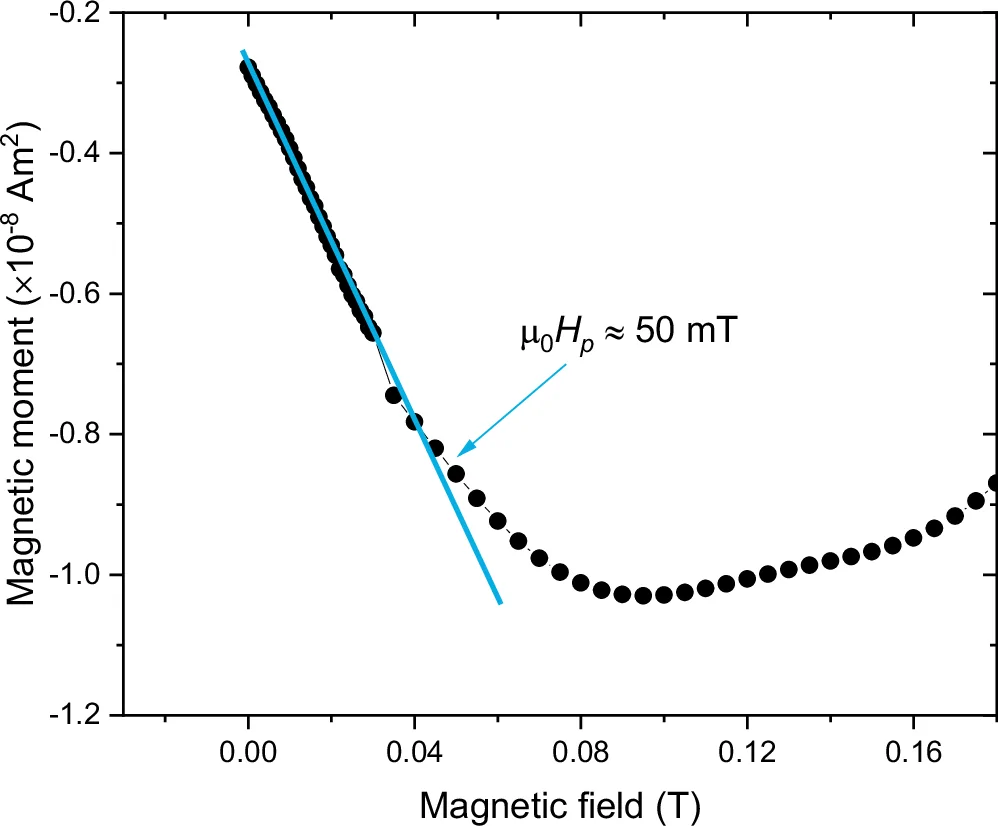
Estimation of μ_0_*H*_*p*_ value for *Im-3m*-H_3_S at *T* = 140 K. Estimation of the μ_0_*H*_*p*_ value from the smoothed virgin curve. The blue line represents the initial linear behavior.

**Fig. 11:**
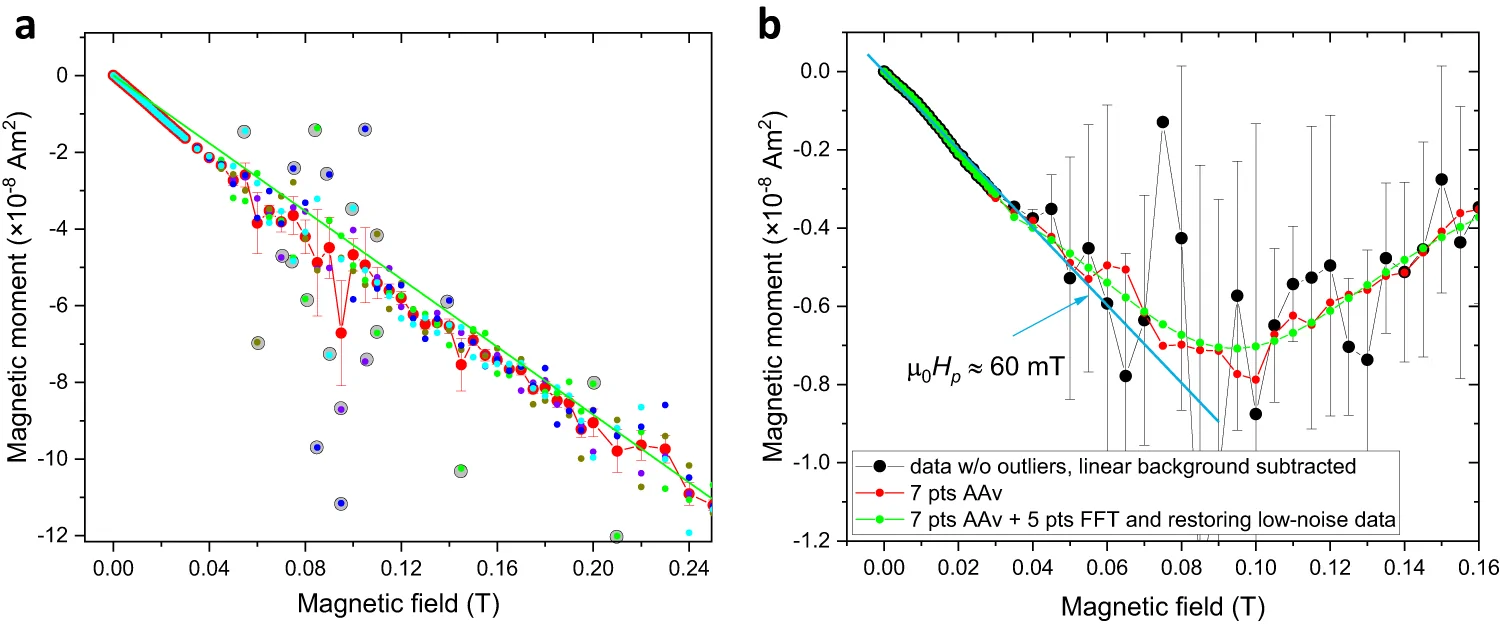
Subtraction of a linear background from the measured *M(H)* data for *Im-3m*-H_3_S at *T* = 120 K, exclusion of outliers, smoothing, and estimation of μ_0_*H*_*p*_. **a** Original measured dataset (red symbols), where each point represents the average of five independent scans (shown as blue, navy, green, purple, and dark yellow circles); error bars indicate the standard deviations. The green line shows the defined linear background. Gray circles mark the excluded outliers identified from the five independent scans. **b** Smoothed *M(H)* dataset obtained using two successive smoothing procedures (AAv and FFT), with the low-noise portion restored. The blue line represents the initial linear behavior used for the μ_0_*H*_*p*_ estimation.

**Fig. 12:**
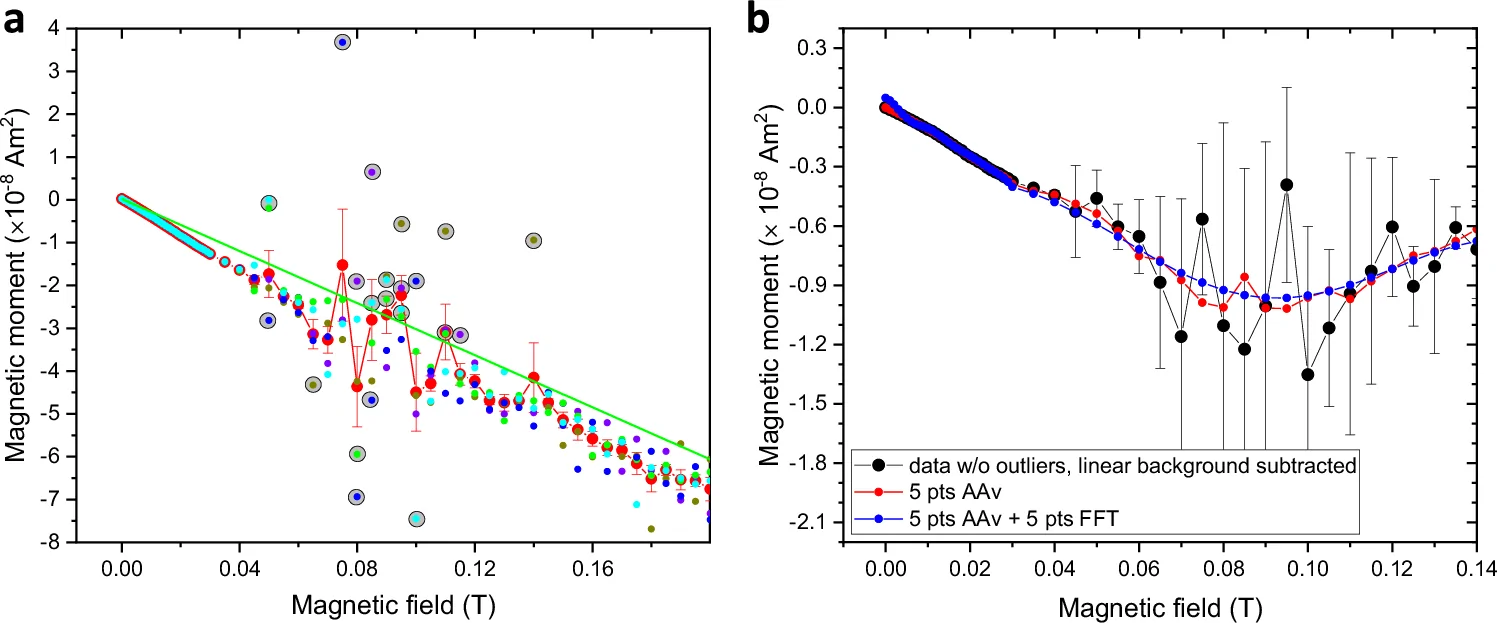
Subtraction of a linear background from the measured *M(H)* data for *Im-3m*-H_3_S at *T* = 40 K and omission of outliers from the high-noise region of the virgin curve. **a** Original measured dataset, where each point represents the average of five independent scans (red symbols); error bars indicate the standard deviations. The green line shows the defined linear background. Gray circles mark the excluded outliers identified from the five independent scans (shown as blue, navy, green, purple, and dark yellow circles). **b** Smoothed *M(H)* dataset obtained using two successive smoothing procedures (AAv and FFT) low-field deviation of the 5 pts AAv + 5 pts FFT curve from the measured data is an artifact of the Smoothing procedure.

**Fig. 13:**
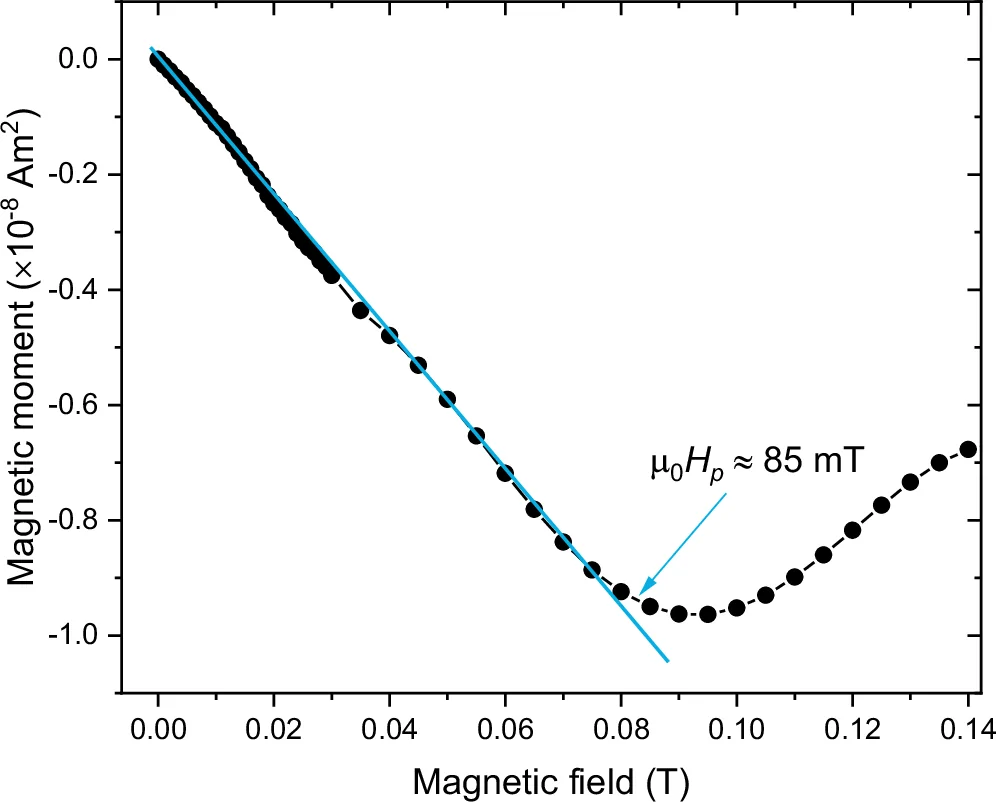
Estimation of μ_0_*H*_*p*_ value *Im-3m*-H_3_S at *T* = 40 K. Estimation of the μ_0_*H*_*p*_ value from the smoothed virgin curve. The blue line represents the initial linear behavior.

**Fig. 14:**
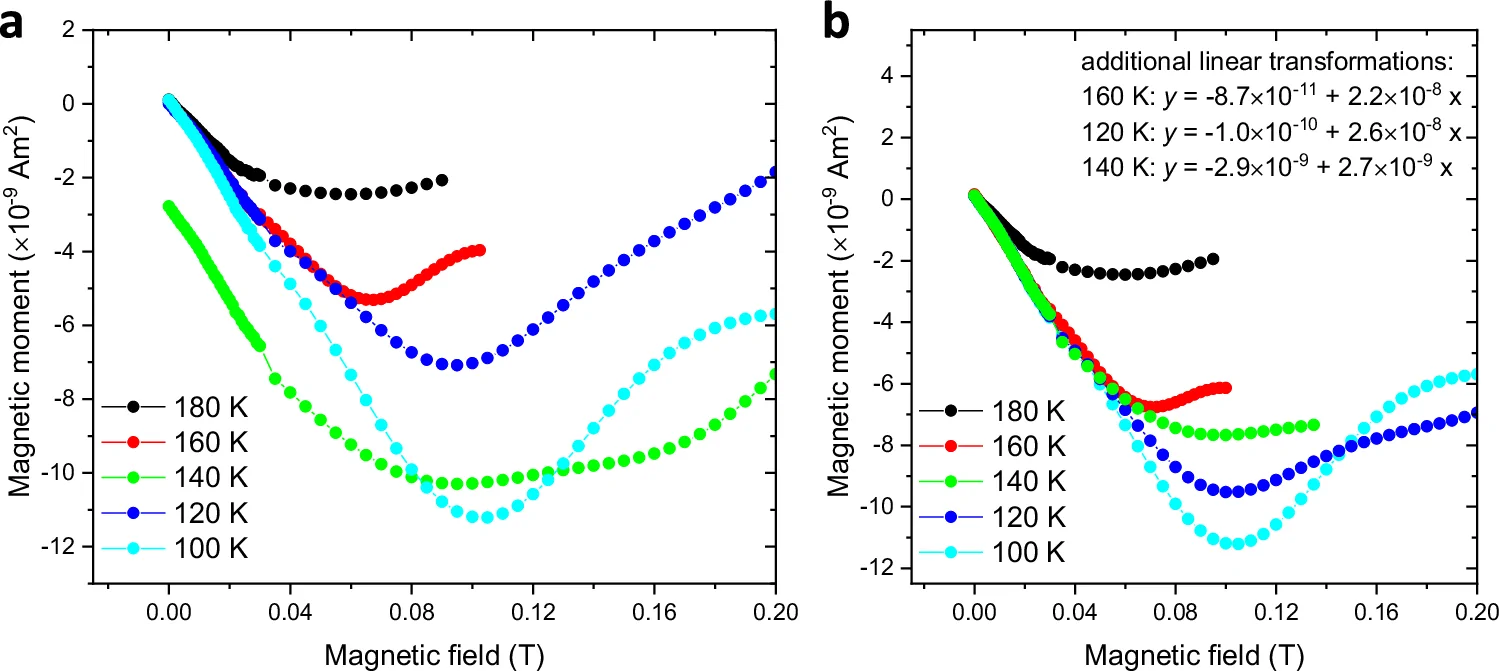
The summary plot for *Im-3m*-H_3_S demonstrating the onset of deviation of the virgin *M(H)* curves from the initial linear trend at different temperatures. **a** Virgin curves measured at different temperatures, smoothed, and background-subtracted using the procedures described above. **b** The same virgin curves after applying additional linear transformations to align their initial linear trends. For this, linear functions were subtracted from the datasets measured at 160 K, 140 K, and 120 K (the exact functions are listed in the upper-right corner).

**Fig. 15:**
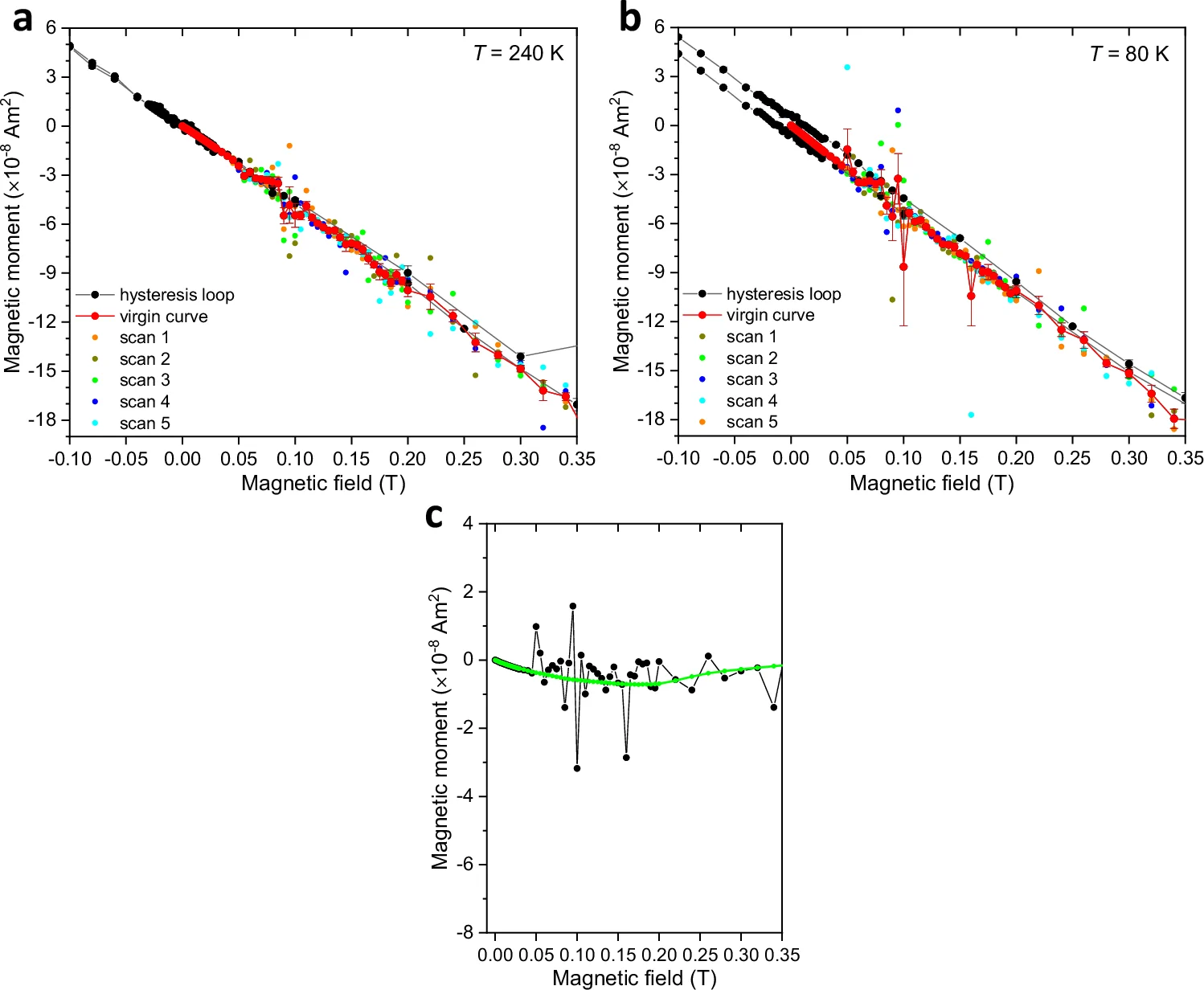
Visualization of the onset of deviation of the virgin *M(H)* curve from the initial linear trend in the *Fm-3m*-LaH_10_ sample. **a**, **b** Hysteresis loops measured by sweeping the applied magnetic field from −1 to 1 Tesla (black circles) and the virgin portion of the *M(H)* data (red circles) at *T* = 240 K and 80 K, respectively. Error bars represent standard deviations estimated from five independent scans for each automatically averaged value (red symbols). The independent scans are plotted as circles of different colors (shown as blue, navy, green, orange, and dark yellow circles). **c** Direct subtraction *M(H, T =* 80 K*)*–*M(H, T =* 240 K*)* without excluding outliers and re-averaging of the data (black symbols). The final averaged/smoothed dataset from the original paper^1^ is shown in green.

## Data Availability

The data supporting the findings of this study are openly available at the Open Science Framework (https://osf.io/7wqxb/) with https://doi.org/10.17605/OSF.IO/7WQXB. Correspondence and requests for materials should be addressed to V.S. Minkov and E.F. Talantsev.
