# Supplementary material for: Magnetic field screening in hydrogen-rich high-temperature superconductors
Source: Nat Commun. 2022 Jun 9;13:3194. doi: 10.1038/s41467-022-30782-x (PMC9184750; doi:10.1038/s41467-022-30782-x)
Supplement: Supplementary file 1 — Supplementary Information [file 41467_2022_30782_MOESM1_ESM.pdf]

Supplementary Information for

Magnetic field screening in hydrogen-rich high-temperature superconductors

V. S. Minkov, S. L. Bud'ko, F. F. Balakirev, V. B. Prakapenka, S. Chariton, R. J. Husband, H. P. Liermann, M. I. Eremets

Correspondence to: m.eremets@mpic.de; v.minkov@mpic.de

This PDF file includes:

Supplementary Notes
Supplementary Figures S1 to S14
Supplementary References

SUPPLEMENTARY NOTES

Estimation of a sample thickness

A thickness of the prepared samples was estimated using optical microscopy. After compression to P_D of ~167 GPa the hole in metal gaskets restraining the samples expanded from ~90 µm to ~180 µm for the sandwich with S and from ~80 µm to ~190 µm for the sandwich with LaH_3 (Figure S13). We estimated the thickness of the final samples by considering *i*) the visual expansion of samples during pressurizing, *ii*) the pressure-induced compressibility of S and LaH_3, and *iii*) the expansion of product volumes after the hydrogenation reaction. The area of the initial S and LaH_3 samples increased by ~3.3 and ~3.9 after pressurizing, respectively. At the same time, a molar volume decreases by ~3 times for S (from ~25.5 Å^3/ S atom at ambient pressure[1] to ~8.7 Å^3/ S atom at 155 GPa[2]) and ~2 times for LaH_3 (from ~44.8 Å^3/ La atom at ambient pressure[3] to ~21.9 Å^3/ La atom at 130 GPa[4,5]). Assuming that the sandwich expands evenly the final thickness of S and LaH_3 plates in the pressurized sandwiches was estimated as ~1.7 µm and ~1.2 µm, respectively. After hydrogenation a molar volume of products increases by ~1.6 times (to ~14.3 Å^3/ S atom in *Im-3m*-H_3S, and ~34.3 Å^3/ La atom in *Fm-3m*-LaH_{10}). Consequently, the thickness of the synthesized H_3S and LaH_{10} samples should be ~2.8 µm and ~1.9 µm, respectively. These estimations are in good agreement with the range of theoretical calculated maximum and minimum values of thickness in these samples (see below).

Estimation of theoretical upper and lower limits of a thickness of samples

The upper limit of a sample thickness can be estimated by taking into account a complete chemical reaction and maximum value of a sample chamber volume realized between diamond anvils at high pressure. Based on Ref[6] the equilibrium thickness of the tungsten gasket at the edge of diamond culets should be ~3 µm for a 90-µm-diameter culet at ~150 GPa and ~2.5 µm for a 75-µm-diameter culet at ~130 GPa. However, the final thickness for much softer materials such as S, NH_3BH_3 and LaH_3 must be even thinner at high pressures. We supposed the final thickness of our samples at the edge of culets as ~2.5 µm and ~2.0 µm for 90-µm- and 75-µm-diameter culets, respectively. Furthermore, we considered an additional contribution to the sample chamber volume due to the cupping deformation of anvils at high pressure, which is significant. According to both computational[7] and experimental[6] studies 75-µm- and 90-µm-diameter culets concave at ~1.5 and ~1.8 µm, respectively. By knowing the cupping effect and final distance between anvils we can calculate the total available volume for a sample and estimate the volume ratio between S or LaH_3 and NH_3BH_3 for a complete conversion into H_3S and LaH_{10}. The extrapolation of the compressibility data for NH_3BH_3 from Ref[8] gives the value of ~22.5 Å^3 per a molecule at ~150 GPa. Assuming complete decomposition of NH_3BH_3 into BN and H_2 on heating, the stoichiometry of chemical reactions, molar volumes of reactants and products we estimated the maximum thickness of H_3S as ~3.1 µm and LaH_{10} as ~2.5 µm.

The lower limit of a sample thickness can be evaluated from the X-ray diffraction experiments. Based on our experience the minimum thickness of sputtered gold leads, which give reasonable intensities in X-ray powder diffraction patterns at the same experimental conditions is ~100 nm. We used these data as a reference for further estimations. The intensity of the diffracted beam is proportional to the square of atomic form factor. Using the literature data for atomic form factors[9] and considering the increase in density of the products at high pressure we estimated the minimum thickness of H_3S as ~2.1 µm and LaH_{10} as ~0.6 µm.

Prior test magnetization measurements

To verify the magnetization measurements for samples housing in the miniature DACs we performed test measurements for the known and well-studied ambient-pressure superconductors. The primary goal of the test measurements was to demonstrate that *i*) superconducting transitions and *ii*) a lower critical field H_{c1} can reliably be detected for a ~100-μm-diameter sample residing in the miniature DAC. For this purpose we used the following materials: single-crystal samples of MgB_2 (synthesized at Ames Laboratory, a size of 35×25×17 μm^3) and $Bi_2Sr_2CaCu_2O_8$ (synthesized at Brookhaven National Laboratory, a size of 100×80×10 μm^3), and powder sample of MgB_2 (Sigma-Aldrich, ≥99%, compacted into a disk with a size of ∅120×10 μm^3 and sandwiched with NH_3BH_3.). The size of these materials was comparable with that of the synthesized *Im-3m*-H_3S and *Fm-3m*-LaH_{10} samples. The whole assembly of the test DACs entirely imitated the conditions for H_3S and LaH_{10} samples under high pressure: the test materials were put into the drilled hole in a rhenium gasket, which was then clamped between two diamond anvils.

Due to the low magnetic signal from the DAC one can unambiguously detect the diamagnetic transition into the superconducting state on the ZFC and FC magnetization curves from the overall magnetic signal without subtraction a background of the DAC (Figure S14). The T_cs in the tested materials inside the miniature DACs were observed at T_c ~38 K for the single-crystal MgB_2 at ambient pressure, T_c ~35 K for the powder MgB_2 at P_D ~1 GPa, and T_c ~88 K for the single-crystal $Bi_2Sr_2CaCu_2O_8$ at ambient pressure. These values are in excellent agreement with the literature data[10-13]. We also performed magnetic measurements using the standard technique and placed the same test samples in a gelatin capsule and fixed them by a thin layer of low viscosity mineral oil. The capsule was then put inside a transparent plastic tube, which was inserted into SQUID. In these corroborating measurements, the diamagnetic character of the superconducting transitions and observed T_c*s* of MgB_2 and $Bi_2Sr_2CaCu_2O_8$ perfectly agree with the measurements in DACs. However, the values of ΔM measured for the test samples in gelatin capsules are smaller because the orientation of single-crystal samples lying onto the hemispherical bottom of capsules differs from that in DACs. The magnetic response strongly depends on the orientation of a sample in an applied magnetic field and is determined by a demagnetization factor. This effect is even more pronounced for the anisotropic layered crystal structures of both MgB_2 and $Bi_2Sr_2CaCu_2O_8$. For instance, the thin-plate shaped single-crystal of $Bi_2Sr_2CaCu_2O_8$ was placed between two anvils in DAC perpendicular to an applied magnetic field and this yielded the maximum value of the measured signal.

The same single-crystal $Bi_2Sr_2CaCu_2O_8$ in the miniature DAC was used for the *M(H)* test magnetization measurements and determination of a H_{c1} value. The *M(H)* data are summarized in Figure S14g. The extrapolation of the $H_p(T)$ data to 0 K gives $H_p(0\ K)$ = 2.5 ± 0.2 mT. To connect the sample magnetic moment with the applied magnetic field we used the effective demagnetizing factor *N*. According to Ref[14] for the cuboid-shaped test crystal N = 0.8556, and thus $H_{c1}(0\ K) = \frac{1}{1-N} H_p(0\ K)$ = 17.3 ± 1.4 mT, what is in good agreement with the literature data[12] (Figure S14i).

SUPPLEMENTARY FIGURES

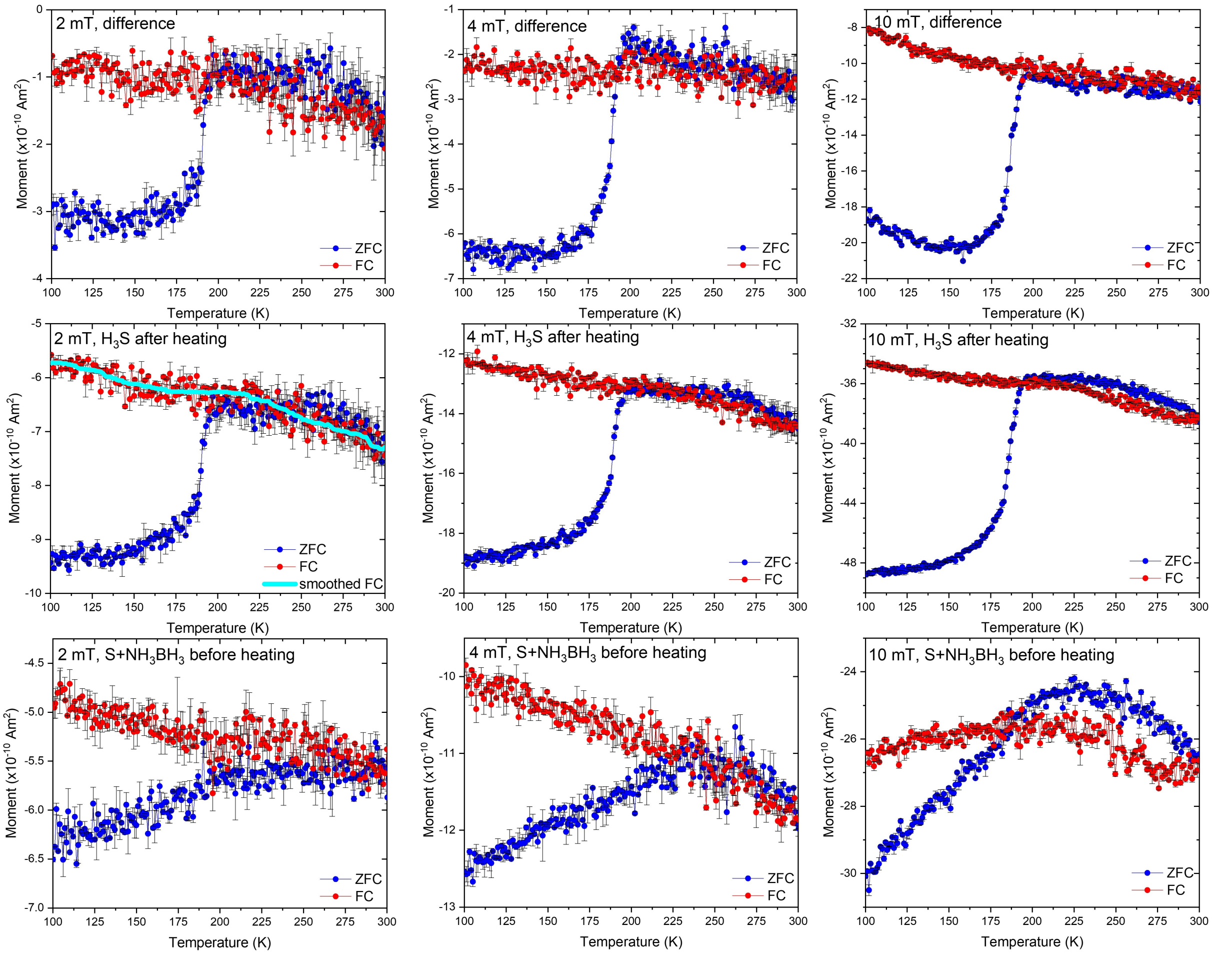

Supplementary Figure S1.

$M(T)$ magnetization data of the sandwiched sample with S and NH_3BH_3 pressurized at $P_D = 167 \pm 7$ GPa at 2, 4 and 10 mT (lower panel), the heated sample with the superconducting *Im-3m*-H_3S phase at $P_S = 155 \pm 5$ GPa ($P_D = 162 \pm 3$ GPa, $P_H = 155$ GPa) at 2, 4, 10 mT (middle panel), and the difference plot (upper panel). The blue and red circles correspond to the zero-field-cooled (ZFC) and filed-cooled (FC) measurements. Light blue smoothed curve shows the subtle Meissner effect in FC measurements at 2 mT. We used percentile filter and 40 points for smoothing the raw data.

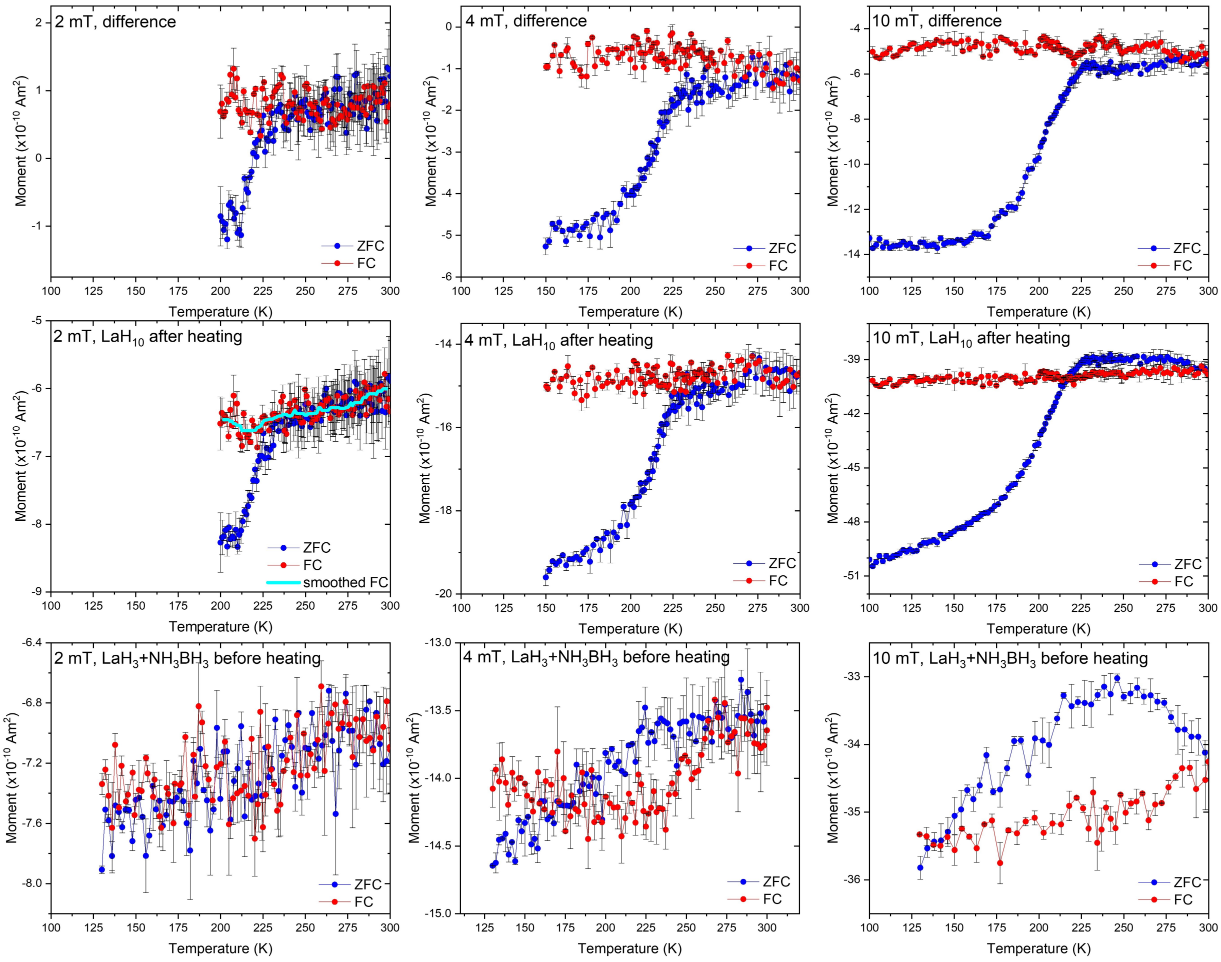

Supplementary Figure S2.

$M(T)$ magnetization data of the sandwiched sample with LaH_3 and NH_3BH_3 pressurized at P_D = 168±7 GPa at 2, 4 and 10 mT (lower panel), the heated sample with the superconducting *Fm-3m*-LaH_{10} phase at P_S = 130±8 GPa (P_D = 157±3 GPa) at 2, 4, 10 mT (middle panel), and the difference plot (upper panel). The blue and red circles correspond to the zero-field-cooled (ZFC) and filed-cooled (FC) measurements. Light blue smoothed curve shows the subtle Meissner effect in FC measurements at 2 mT. We used percentile filter and 16 points for smoothing the raw data.

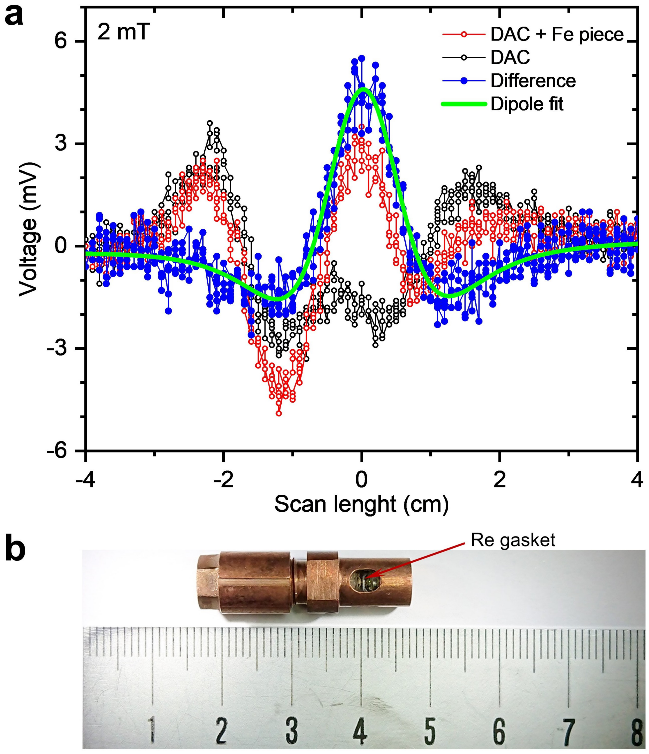

Supplementary Figure S3.

Centering of a sample residing in the miniature DAC using a ferromagnetic signal of the steel piece with a size of 140×100×25 μm^3 attached to the rhenium gasket. **a**) The red, black and blue circles and green curve correspond to the voltage curves of DC scans of the miniature DAC with and without steel piece, the difference, and dipole fit, respectively. Here is the centering of the sample with the *Fm-3m*-LaH_{10} phase in DAC at 295 K and 2 mT. **b**) Photo of the miniature DAC scaled to the length of DC scans in panel **a**. The asymmetric design of the DAC causes asymmetric profiles of voltage curves of DC scans (black and red curves).

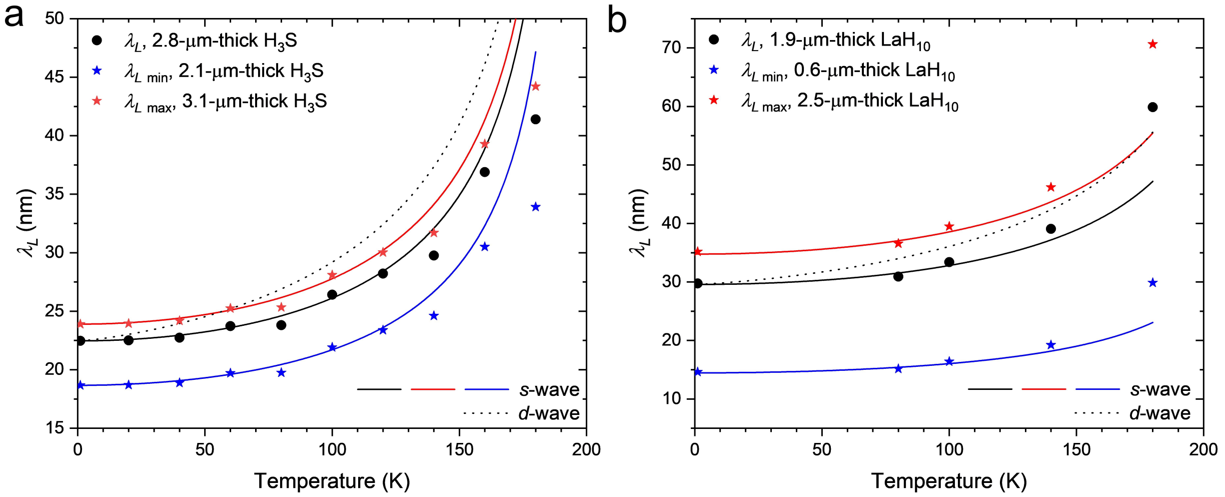

Supplementary Figure S4.

Temperature-dependence of the London penetration depth, λ_L, of *Im-3m*-H_3S at P_S= 155±5 GPa, **a**, and *Fm-3m*-LaH_{10} at P_S= 130±8 GPa, **b**. The black circles correspond to the λ_L values, which were determined using the sample thickness of 2.8 µm for H_3S and 1.9 µm for LaH_{10} samples estimated from the optical microscopy data. The red and blue stars correspond to maximum and minimum λ_L values, which were estimated using the theoretical calculated maximum and minimum values of sample thickness. The solid and dotted curves are approximate functional forms for the London penetration depth in the entire temperature range for the *s*-wave ($\lambda_L(T) = \lambda_L(0\ K)/\sqrt{1-t^2}$, where $t = T/T_c$) and *d*-wave ($\lambda_L(T) = \lambda_L(0\ K)/\sqrt{1-t^{4/3}}$) clean limits.

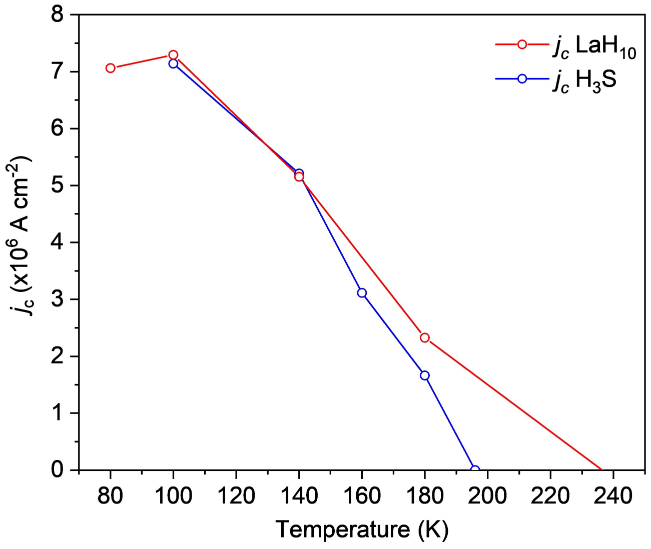

Supplementary Figure S5.

Temperature-dependence of the critical current density j_c in the *Im-3m*-H_3S and *Fm-3m*-LaH_{10} samples estimated from the hysteretic loops of *M(H)* data using Bean´s model.

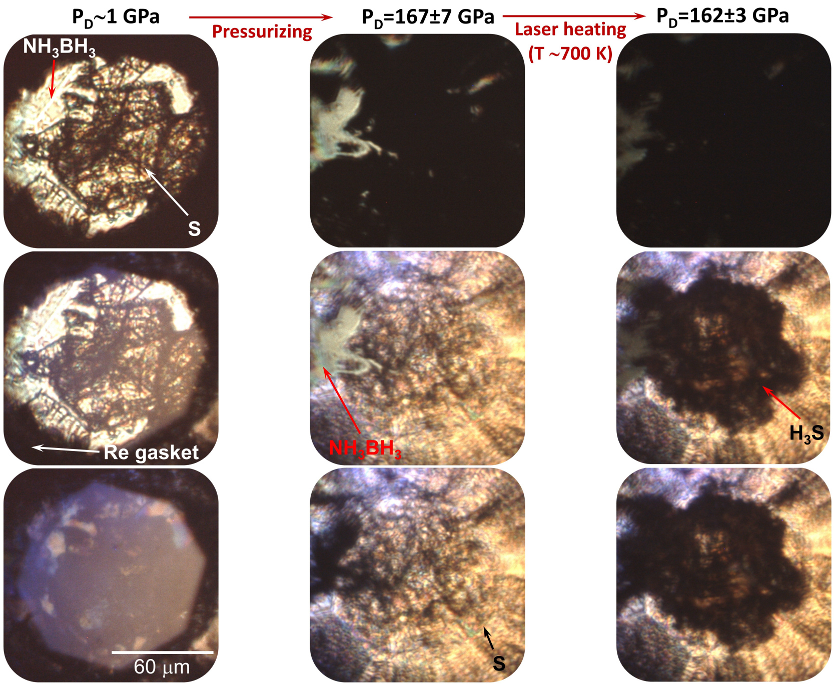

Supplementary Figure S6.

Photos of the sandwiched sample with S and NH_3BH_3 after clamping at P_D ~1 GPa (left panel) and pressurizing to P_D = 167±7 GPa (central panel), and the heated sample with the superconducting *Im-3m*-H_3S phase at P_S = 155±5 GPa (P_D = 162±3 GPa, P_H = 155 GPa). The photos are taken in transmitted (top), transmitted and reflected (center), and reflected (bottom) light.

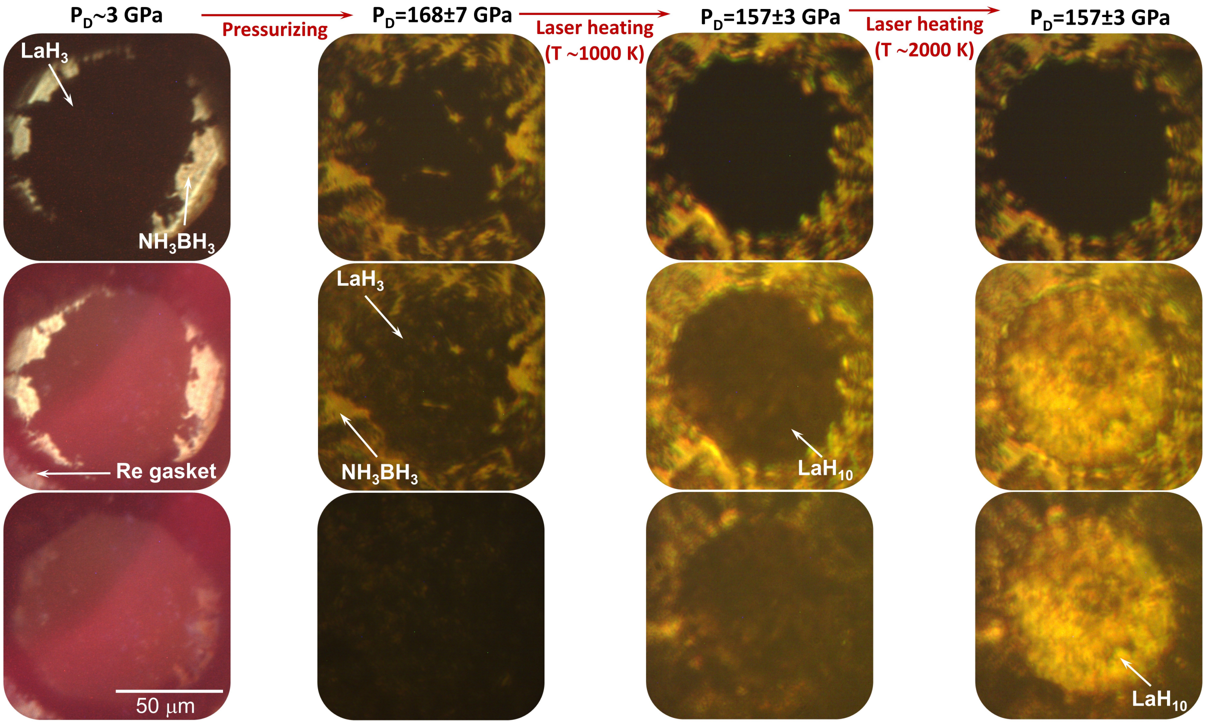

Supplementary Figure S7.

Photos of the sandwiched sample with LaH_3 and NH_3BH_3 after clamping at P_D~3 GPa (left panel) and pressurizing to P_D = 168±7 GPa (second panel), and the sample with the superconducting *Fm-3m*-LaH_{10} phase at P_S = 130±8 GPa (P_D = 157±3 GPa) after heating by a pulse laser at ~1000 K (third panel) and ~2000 K (right panel). The photos are taken in transmitted (top), transmitted and reflected (center), and reflected (bottom) light.

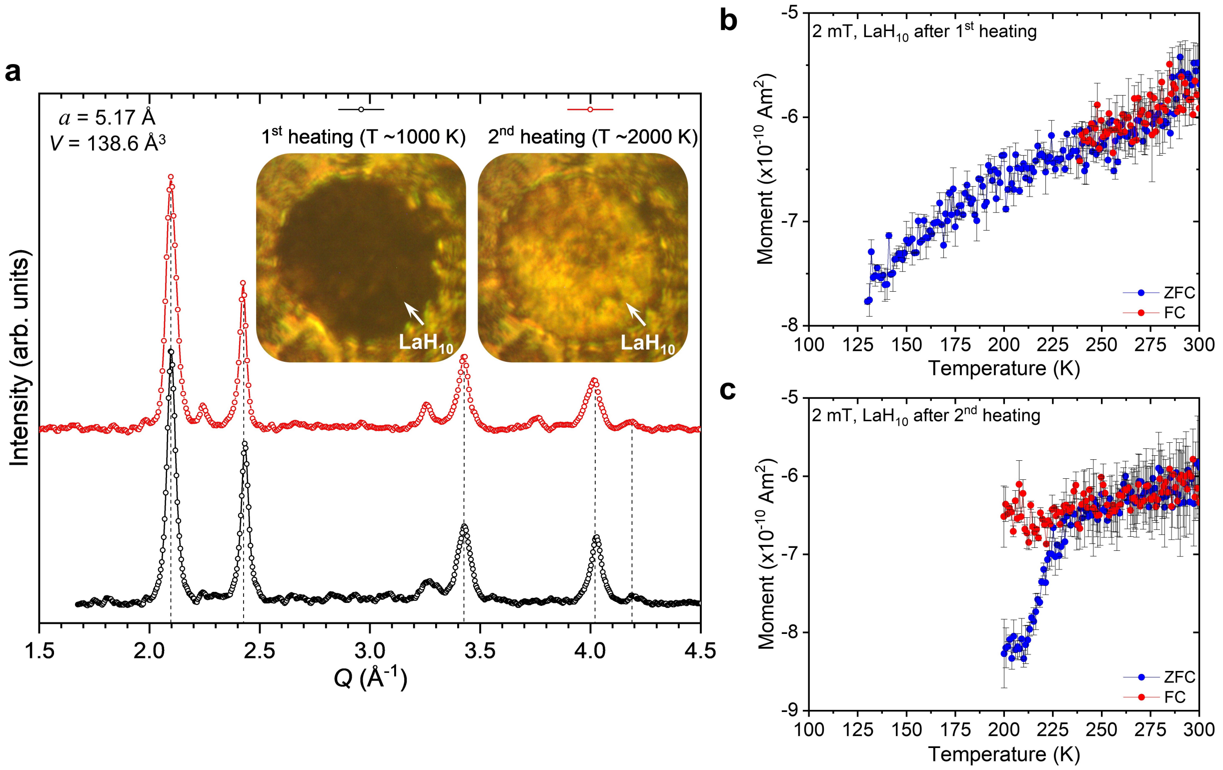

Supplementary Figure S8.

Characterization of the sample of the *Fm-3m*-LaH_{10} phase after two laser heating treatments at ~1000 K and ~2000 K. **a**) X-ray powder diffraction patterns collected from the same spot of the sample after the first and second heating. Positions of peaks of the *Fm-3m*-LaH_{10} phase are almost the same after both high-temperature treatments (marked by dashed vertical lines). Insets are photos of the heated sample taken in transmitted and reflected light. Sample became shinier after the second treatment at ~2000 K. **b**) and **c**) $M(T)$ magnetization data of the sample of *Fm-3m*-LaH_{10} after the first and second heating, respectively.

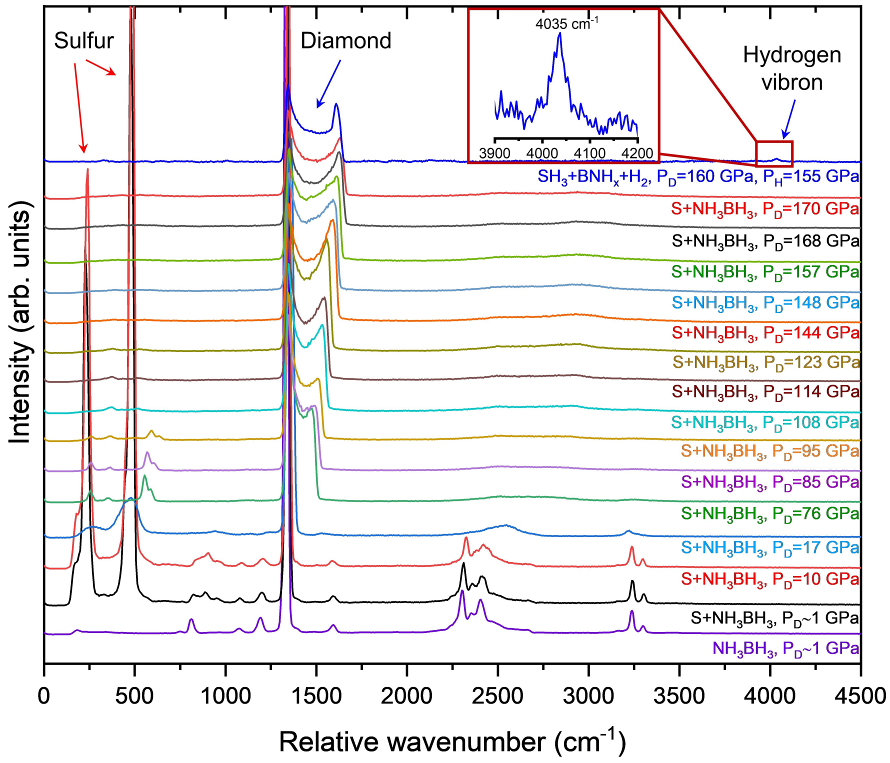

Supplementary Figure S9.

Raman spectra of the sandwiched sample with S and NH_3BH_3 upon pressurizing from P_D ~1 GPa (black Raman spectrum) to P_D = 167±7 GPa (red Raman spectrum), demonstrating the metallization of sulfur. The blue Raman spectrum on the top corresponds to the sample with the superconducting *Im-3m*-H_3S phase, which was synthesized after heating at ~700 K by a pulse laser. The wavenumber of the hydrogen vibron, which was appeared in the Raman spectrum after heating of NH_3BH_3, corresponds to a pressure value of P_H = 155 GPa, slightly lower than P_D = 162±3 GPa estimated by the diamond scale[15].

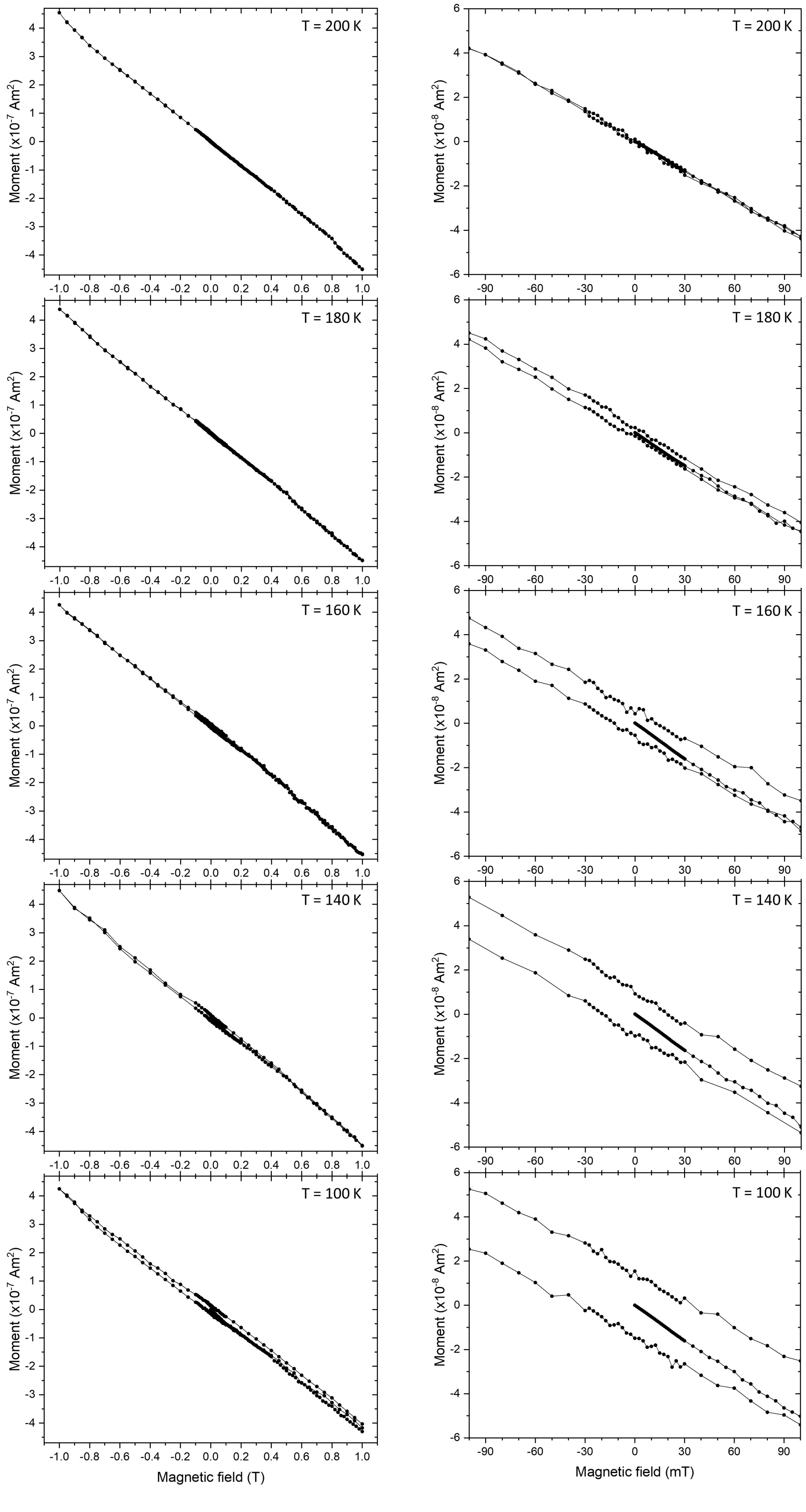
T = 200 K
T = 180 K
T = 160 K
T = 140 K
T = 100 K
Moment (x10^{-7} Am^2)
Moment (x10^{-8} Am^2)
Magnetic field (T)
Magnetic field (mT)

Supplementary Figure S10.

M(*H*) magnetization data of the heated sample with the *Im-3m*-H_3S at P_S= 155±5 GPa (P_D= 162±3 GPa, P_H= 155 GPa) at several temperatures: in a normal metallic state above T_c at 200 K, and in a superconducting state below T_c at 180, 140, 100 and 80 K. The left panel shows the full range of hysteresis and the right panel shows the enlarged magnetic field range of -0.1 – 0.1 T.

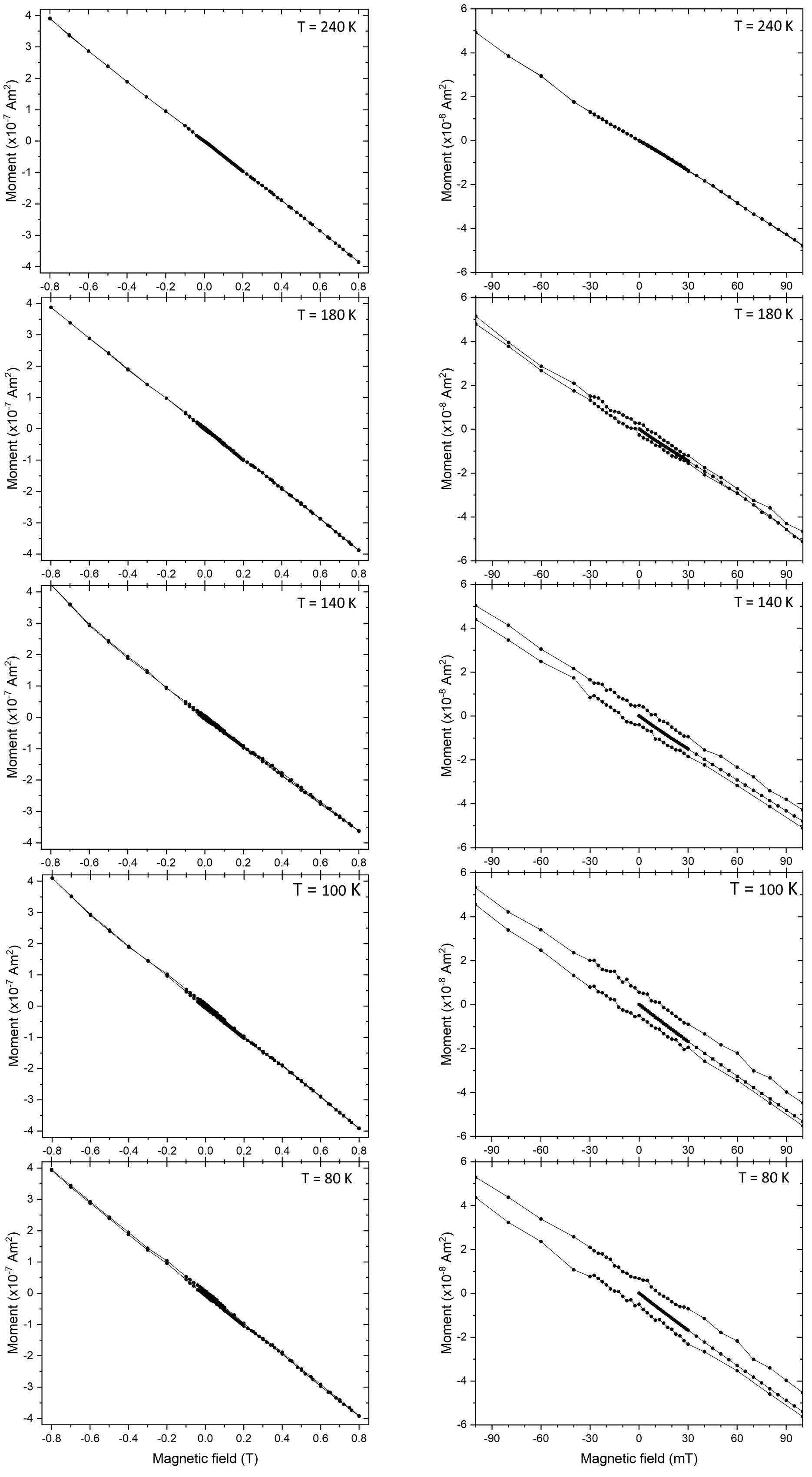
T = 240 K
T = 180 K
T = 140 K
T = 100 K
T = 80 K
Moment (x10^{-7} Am^2)
Moment (x10^{-8} Am^2)
Magnetic field (T)
Magnetic field (mT)

Supplementary Figure S11.

$M(H)$ magnetization data of the heated sample with the $Fm\text{-}3m$-LaH_{10} phase at P_S = 130±8 GPa (P_D = 157±3 GPa) at several temperatures: in a normal metallic state above T_c at 240 K, and in a superconducting state below T_c at 180, 140, 100 and 80 K. The left panel shows the full range of hysteresis and the right panel shows the enlarged magnetic field range of -0.1 – 0.1 T.

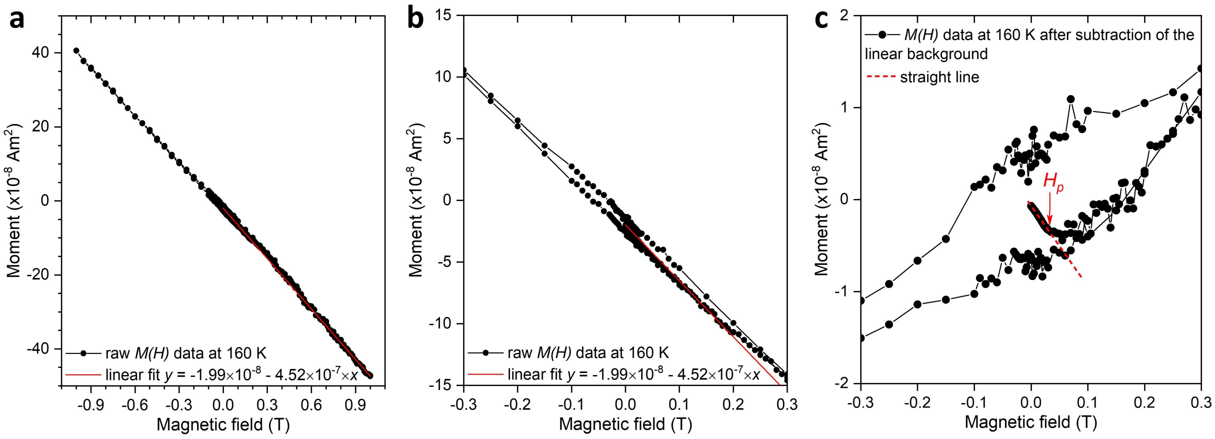

Supplementary Figure S12.

Subtraction of the linear background for the better illustration of the value of H_p, at which an applied magnetic field starts to penetrate into the sample. **a**, **b** Raw *M(H)* magnetization data measured at T = 160 K (black circles) and the linear background, which was determined as the straight line connecting two endpoints: the magnetic moment value at H = 0 T (the starting point of measurements) and the magnetic moment value at H = 1 T (the highest value of the applied magnetic field). **c** Corrected *M(H)* magnetization data after subtraction of the linear background (black circles). The value of H_p, at which an applied magnetic field starts to penetrate into the sample, was determined as the onset of the evident deviation of the *M(H)* data from the linear dependence (red dashed straight line).

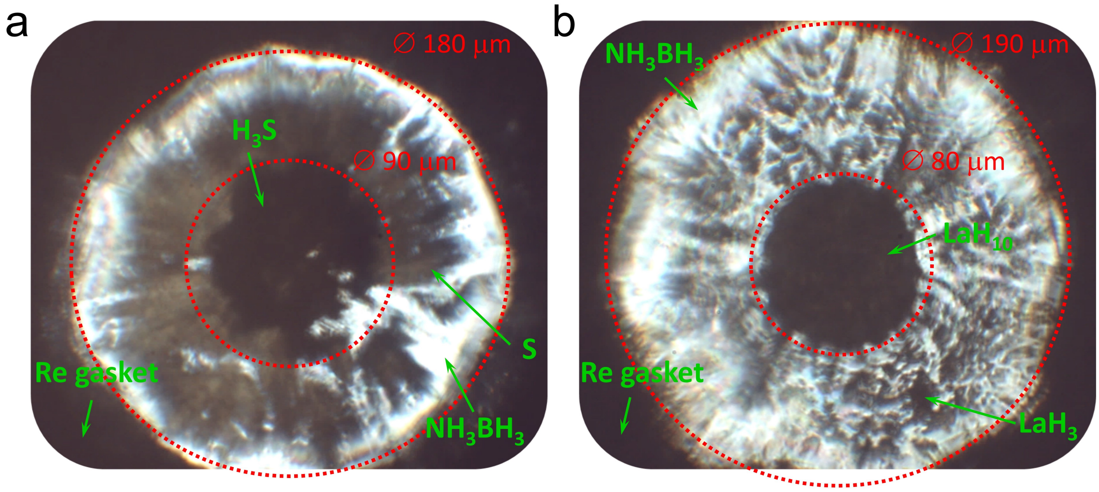

Supplementary Figure S13.

Estimation of a thickness of the superconducting samples in the miniature DACs at high pressure. **a** and **b**, Expansion of the hole in the rhenium gasket restraining the sandwiched samples with H_3S at P_S = 155±5 GPa and LaH_{10} at P_S = 130±8 GPa, respectively. The inner red circles show the starting size of the hole in the rhenium gasket at P_D ~1 and ~3 GPa. Photos are taken with 90%-transmitting and 10%-reflecting light.

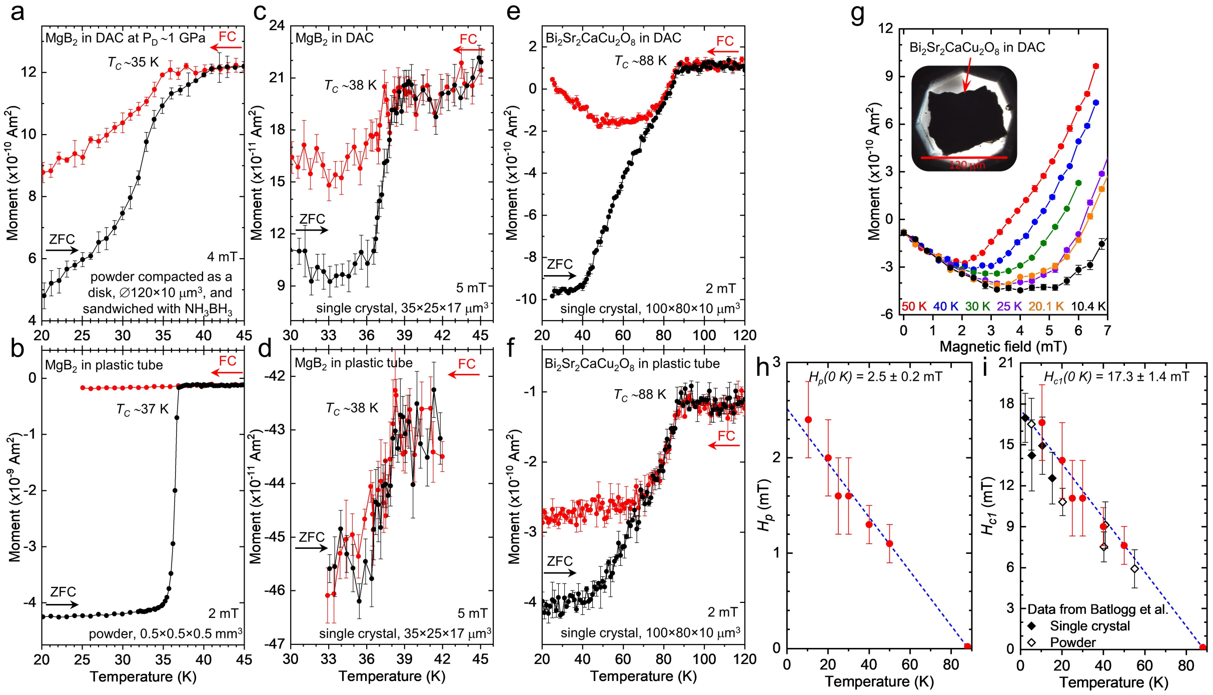

Supplementary Figure S14.

Magnetic measurements of the test materials in a SQUID. *M(T)* magnetization data for powder MgB_2 sample, **a** and **b**, single crystal MgB_2 sample, **c** and **d**, and single crystal $Bi_2Sr_2CaCu_2O_8$ sample, **e** and **f**, housing in the miniature DACs and the standard nonmagnetic plastic tube. Black and red cycles correspond to ZFC and FC magnetization curves. **g**, *M(H)* magnetization data of the single crystal $Bi_2Sr_2CaCu_2O_8$ sample in the DAC at different temperatures. **h** and **i**, $H_p(T)$ and $H_{c1}(T)$ plots of the single crystal $Bi_2Sr_2CaCu_2O_8$ sample in the DAC built from the *M(H)* data without effective demagnetizing factor N and with the estimated $N = 0.8556$, respectively. Red circles and black rhombuses are the present data and the data from Ref[12], respectively.

SUPPLEMENTARY REFERENCES

1. Abrahams, S. The crystal and molecular structure of orthorhombic sulfur. *Acta Crystallographica* **8**, 661-671, doi:doi:10.1107/S0365110X55002089 (1955).
2. Minkov, V. S., Prakapenka, V. B., Greenberg, E. & Eremets, M. I. A Boosted Critical Temperature of 166 K in Superconducting D_3S Synthesized from Elemental Sulfur and Hydrogen. *Angewandte Chemie International Edition* **59**, 18970-18974, doi:https://doi.org/10.1002/anie.202007091 (2020).
3. Palasyuk, T. & Tkacz, M. High-pressure studies of LaH3−δ (δ=0.00, 0.15). *Journal of Alloys and Compounds* **468**, 191-194, doi:https://doi.org/10.1016/j.jallcom.2008.01.054 (2009).
4. Drozdov, A. P. *et al.* Superconductivity at 250 K in lanthanum hydride under high pressures *Nature* **569** 528 (2019).
5. Sun, D. *et al.* High-temperature superconductivity on the verge of a structural instability in lanthanum superhydride. *Nature Communications* **12**, 6863, doi:10.1038/s41467-021-26706-w (2021).
6. Li, B. *et al.* Diamond anvil cell behavior up to 4 Mbar. *Proceedings of the National Academy of Sciences* **115**, 1713, doi:10.1073/pnas.1721425115 (2018).
7. Adams, D. M. & Shaw, A. C. A computer-aided design study of the behaviour of diamond anvils under stress. *Journal of Physics D: Applied Physics* **15**, 1609-1635, doi:10.1088/0022-3727/15/9/006 (1982).
8. Nakano, S. *et al.* Observation of Dihydrogen Bonds in High-Pressure Phases of Ammonia Borane by X-ray and Neutron Diffraction Measurements. *Inorganic Chemistry* **60**, 3065-3073, doi:10.1021/acs.inorgchem.0c03345 (2021).
9. Brown, P. J., Fox, A. G., Maslen, E. N., O'Keefe, M. A. & Willis, B. T. M. *"International Tables for Crystallography"*. Vol. C 554-595 (2006).
10. Saito, E. *et al.* Pressure dependence of T_c in the MgB_2 super- conductor as probed by resistivity measurements. *Journal of Physics: Condensed Matter* **13**, L267-L270, doi:10.1088/0953-8984/13/12/102 (2001).
11. Lorenz, B., Meng, R. L. & Chu, C. W. High-pressure study on MgB_2. *Physical Review B* **64**, 012507, doi:10.1103/PhysRevB.64.012507 (2001).
12. Batlogg, B., Palstra, T. T. M., Schneemeyer, L. F., Van Dover, R. B. & Cava, R. J. Superconducting and normal state parameters of $Bi_{2.2}Sr_2Ca_{0.8}Cu_2O_{8+\delta}$ single crystals: A comparison with $Ba_2YCu_3O_7$. *Physica C: Superconductivity* **153-155**, 1062-1066, doi:https://doi.org/10.1016/0921-4534(88)90200-6 (1988).
13. Mou, D. *et al.* Isotope effect on electron-phonon interaction in the multiband superconductor MgB_2. *Physical Review B* **93**, 144504, doi:10.1103/PhysRevB.93.144504 (2016).
14. Prozorov, R. & Kogan, V. G. Effective Demagnetizing Factors of Diamagnetic Samples of Various Shapes. *Physical Review Applied* **10**, 014030, doi:10.1103/PhysRevApplied.10.014030 (2018).
15. Eremets, M. I. Megabar high-pressure cells for Raman measurements. *Journal of Raman Spectroscopy* **34**, 515 (2003).
