# Supplementary material for: Addendum: Magnetic field screening in hydrogen-rich high-temperature superconductors
Source: Nat Commun. 2026 Oct 6;17:10452. doi: 10.1038/s41467-026-73705-w (PMC13642310; doi:10.1038/s41467-026-73705-w)
Supplement: Supplementary file 1 [file 41467_2026_73705_MOESM1_ESM.pdf]

Supplementary Information for

Addendum: Magnetic field screening in hydrogen-rich high-temperature superconductors

V. S. Minkov[1*], E. F. Talantsev[2,3*], S. L. Bud'ko[4,5], F. F. Balakirev[6], M. I. Eremets[1]

[1]*Max Planck Institute for Chemistry; Hahn Meitner Weg 1, Mainz 55128, Germany*
[2]*M.N. Mikheev Institute of Metal Physics, Ural Branch of the Russian Academy of Sciences, S. Kovalevskoy St 18, 620108 Ekaterinburg, Russian Federation*
[3]*NANOTECH Centre, Ural Federal University, Mira St 19, 620002 Ekaterinburg, Russian Federation*
[4]*Ames National Laboratory, U.S. Department of Energy, Iowa State University; Ames, IA 50011, United States*
[5]*Department of Physics and Astronomy, Iowa State University; Ames, IA 50011, United States*
[6]*Los Alamos National Laboratory; Los Alamos, NM 87545, USA*
*Corresponding authors:
Email: v.minkov@mpic.de (V.S.M); evgeny.talantsev@imp.uran.ru (E.F.T.)

This PDF document includes:

Supplementary Figures S1 to S4

Supplementary References

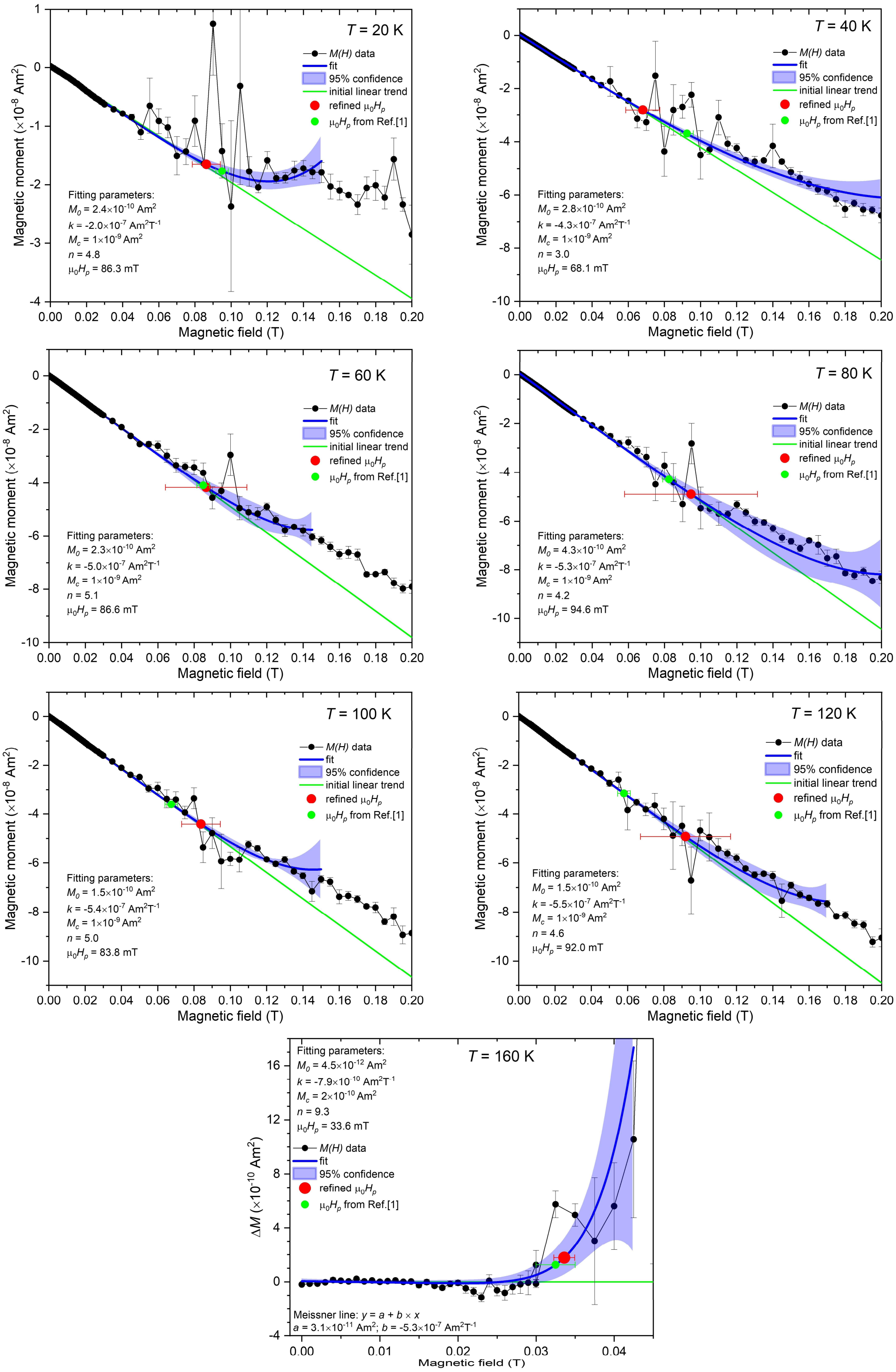

Supplementary Figure S1. The extraction of μ_0H_p from *M(H)* data of the *Im-3m*-H_3S sample at P_S = 155±5 GPa. Black circles represent the measured *M*(*H*) data; red and green circles represent μ_0H_p values estimated in the present *Addendum* and in the original paper[1], respectively. The green line depicts the initial Meissner linear trend of the virgin curve in the Meissner state. This Meissner line is defined as linear fit of the data measured at low applied magnetic fields (≤ 30 mT) and serves as a guide for the eye. Blue curves represent fits with 95% confidence bands.

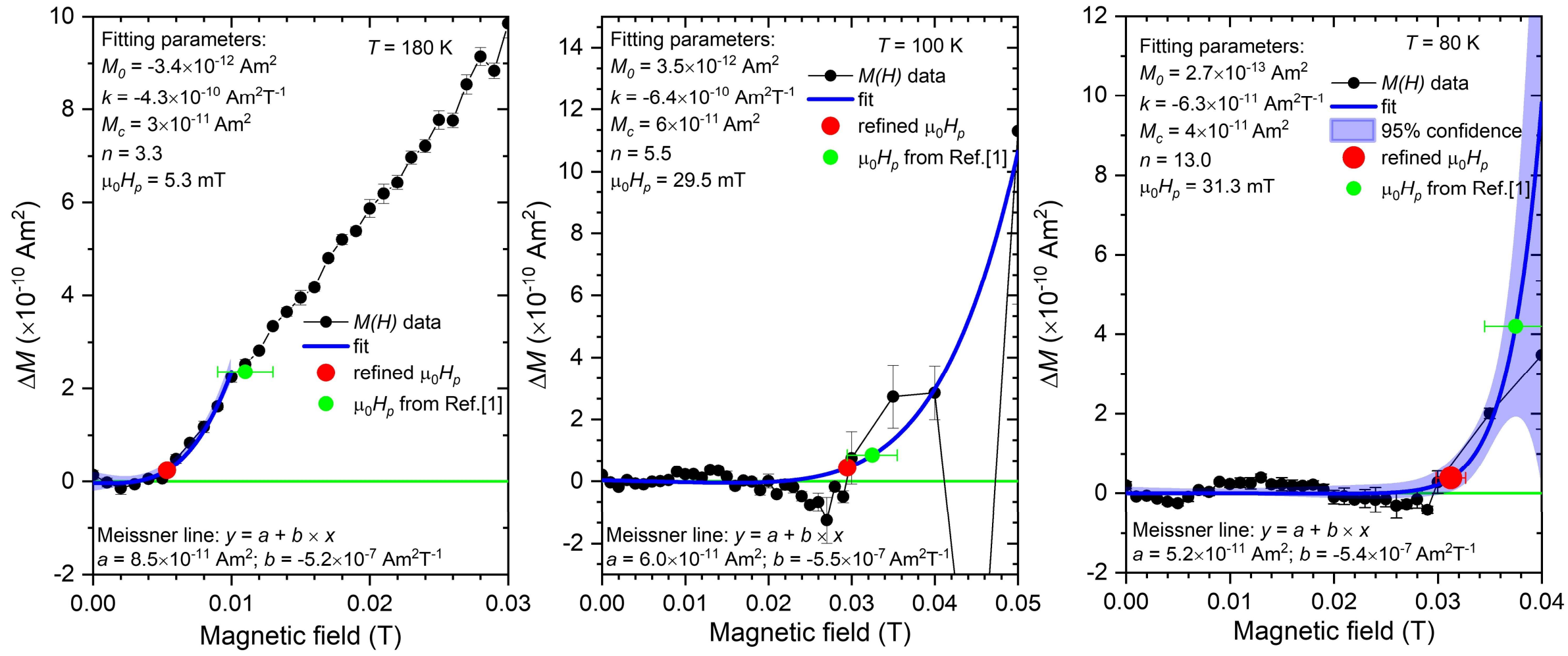

Supplementary Figure S2. The extraction of μ_0H_p from *M(H)* data of the *Fm-3m*-LaH_{10} sample at P_S = 130±8 GPa in the low-noise region of applied magnetic fields (≤ 30 mT). We subtracted the linear function $y = a + b \times x$ (with a and b parameters shown below each curve) from the original $M(H)$ data for better presentation of the initial linear trend of the virgin curve. Black circles represent the measured $M(H)$ data; red and green circles represent μ_0H_p values estimated in the present *Addendum* and in the original paper[1], respectively. The green line depicts the Meissner line ($y = 0$), and blue curves represent the fits with 95% confidence bands.

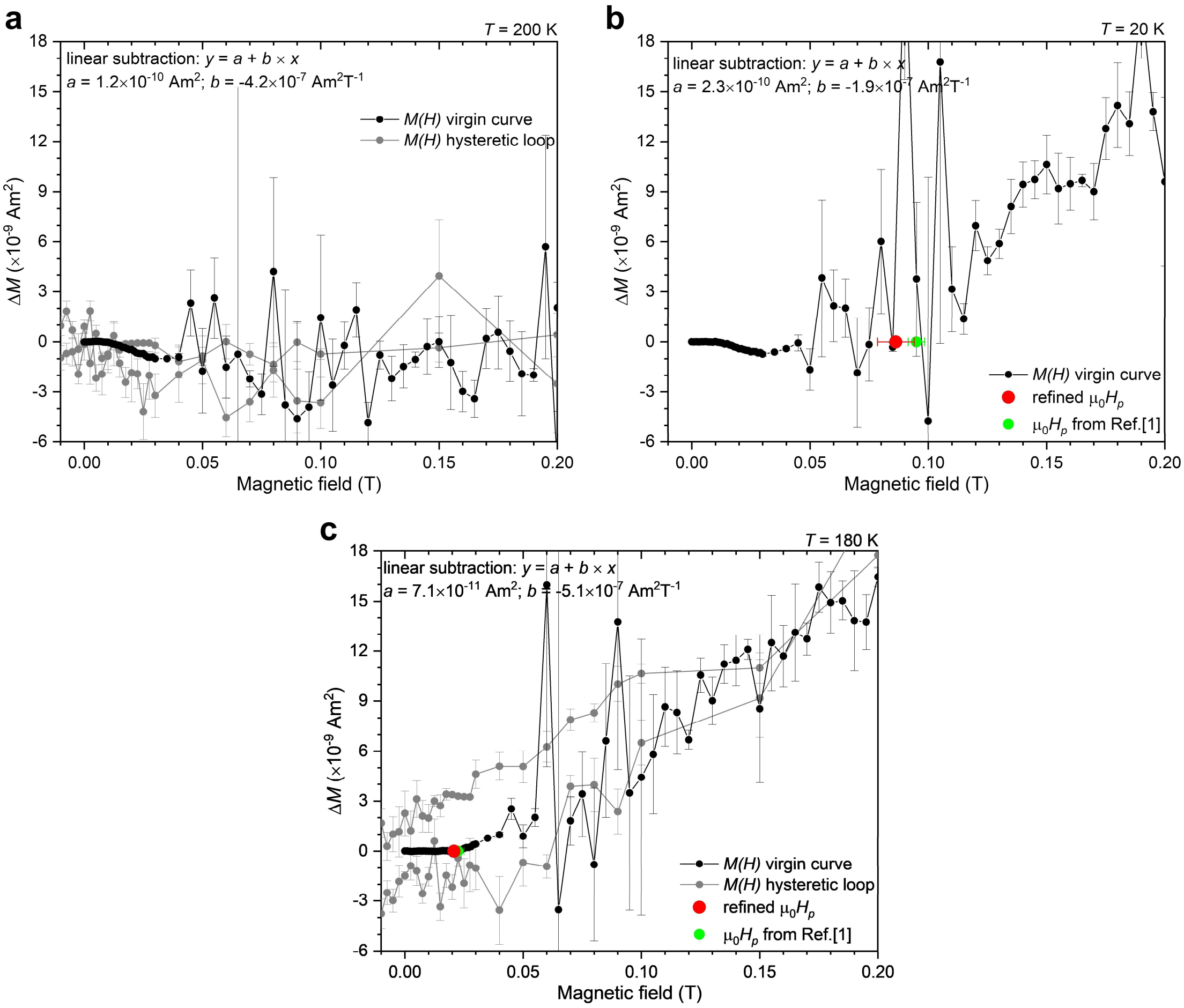

Supplementary Figure S3. Nonlinearity of the background of the diamond anvil cell with the *Im-3m*-H_3S sample at P_S = 155±5 GPa in the region of applied magnetic fields below 30 mT. (**a**) *M(H)* data measured at T = 200 K (above T_c of the superconducting H_3S sample) characterize the DAC background, showing a subtle downturn at approximately 10 mT. Importantly, this effect has the opposite sign compared to the penetration of magnetic flux into the superconducting sample at temperatures below T_c. (**b**) *M(H)* data measured at T = 20 K demonstrate exactly the same downturn from the DAC background, preceding the entrance of magnetic flux into the sample. This indicates that the μ_0H_p value lies beyond 30 mT. (**c**) *M(H)* data measured at T = 180 K indicate that the onset of magnetic flux penetration into the sample occurs at low applied magnetic fields (below 30 mT). In this case, the DAC background does not significantly affect the determination of the μ_0H_p value. To better demonstrate the nonlinear behaviour of the DAC at low magnetic fields, we subtracted the linear background from the measured *M(H)* data. The coefficients of the function $y = a + b \times x$ are obtained by linear fit to the very initial portion of the virgin *M(H)* dataset ($0 < \mu_0H_{appl} < 10$ mT) and are provided in each panel. Black circles represent the virgin portion of the *M*(*H*) data; grey circles correspond to the hysteretic portion of the *M(H)* data; green and red circles correspond to the refined values of μ_0H_p (where applicable).

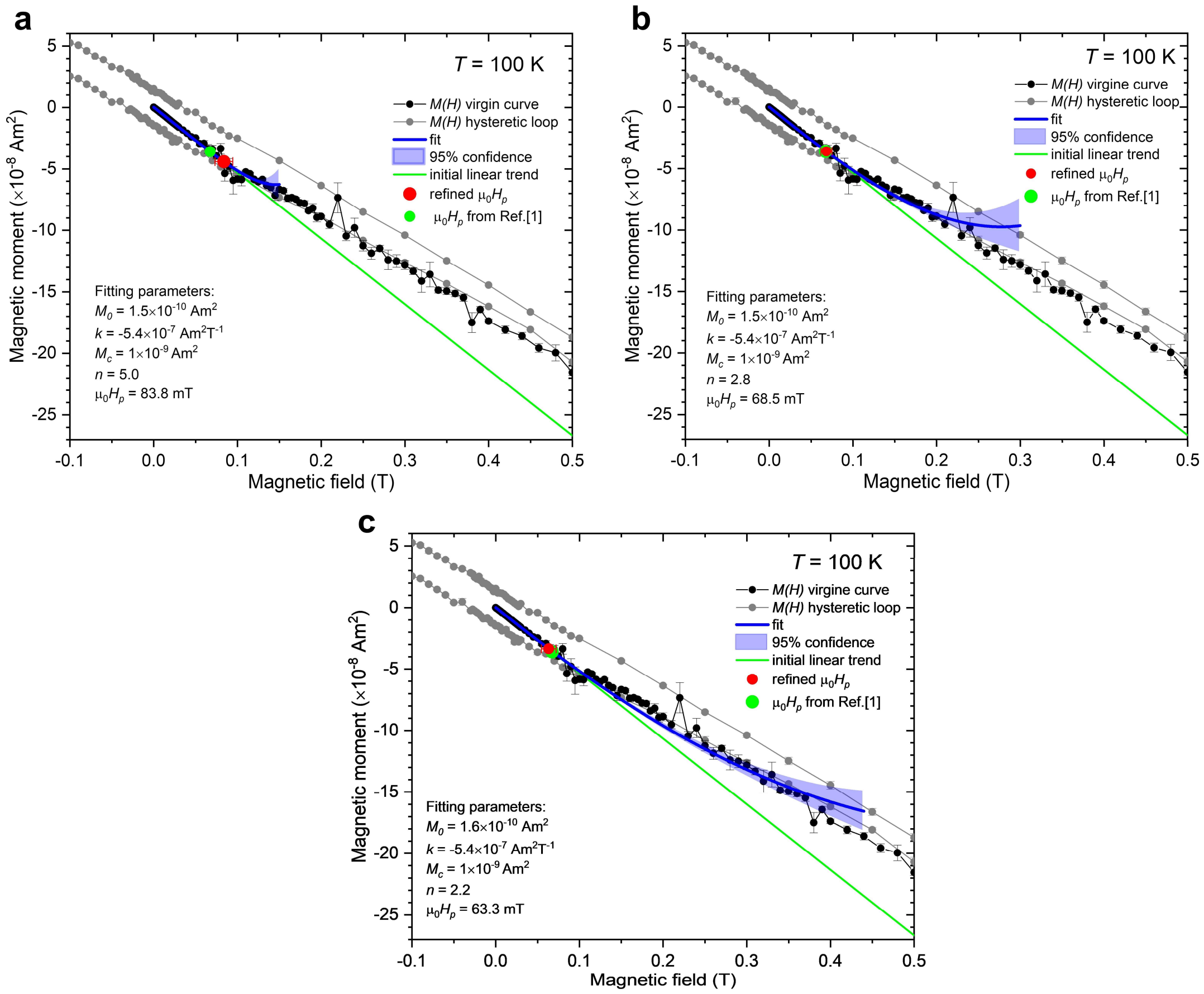

Supplementary Figure S4. Influence of the range of the *M(H)* dataset of the *Im-3m*-H_3S sample (P_S = 155±5 GPa) measured at T = 100 K on $\mu_0 H_p$ values upon fitting (when $\mu_0 H_p$ lies in the region of applied magnetic fields above 30 mT). (**a**) The fitting of the virgin curve of the *M(H)* data is limited by $\mu_0 H_{appl}$ = 150 mT, at which the difference between the measured $M(H_{appl})$ and the Meissner line reaches approximately ten times the value of M_c. The refined $\mu_0 H_p$ = 84±10 mT. (**b**) The fitting of the virgin *M(H)* data is extended to double the range, limited by $\mu_0 H_{appl}$ = 300 mT. In this case, the refined $\mu_0 H_p$ = 68±6 mT. (**c**) The results of the fitting when the selected *M(H)* dataset is limited by $\mu_0 H_{appl}$ = 440 mT. The refined $\mu_0 H_p$ = 63±9 mT. Black circles represent the measured *M*(*H*) data of the virgin magnetization curve; grey circles represent the hysteretic portion of the *M(H)* data; red and green circles represent $\mu_0 H_p$ values estimated in the present *Addendum* and in the original paper[1], respectively. The green line refers to the initial linear trend (Meissner line), and blue curves represent the fits with 95% confidence bands.

Supplementary References

1. Minkov V. S., *et al.* Magnetic field screening in hydrogen-rich high-temperature superconductors. *Nat. Commun.* **13**, 3194 (2022).
